# Peptidase Inhibitor 15 (PI15) Regulates Chlamydial CPAF Activity

**DOI:** 10.3389/fcimb.2018.00183

**Published:** 2018-05-30

**Authors:** Bhupesh K. Prusty, Suvagata R. Chowdhury, Nitish Gulve, Thomas Rudel

**Affiliations:** Biocenter, Chair of Microbiology, University of Würzburg, Würzburg, Germany

**Keywords:** chlamydia, CPAF activation, peptidase inhibitor PI15, chlamydial inclusion, chlamydia serine proteases

## Abstract

Obligate intracellular pathogenic *Chlamydia trachomatis* express several serine proteases whose roles in chlamydial development and pathogenicity are not completely understood. The chlamydial protease CPAF is expressed during the replicative phase of the chlamydial developmental cycle and is secreted into the lumen of the *Chlamydia*-containing vacuole called inclusion. How the secreted protease is activated in the inclusion lumen is currently not fully understood. We have identified human serine peptidase inhibitor PI15 as a potential host factor involved in the regulation of CPAF activation. Silencing expression as well as over expression of PI15 affected normal development of *Chlamydia*. PI15 was transported into the chlamydial inclusion lumen where it co-localized with CPAF aggregates. We show that PI15 binds to the CPAF zymogen and potentially induces CPAF protease activity at low concentrations. However, at high concentrations PI15 inhibits CPAF activity possibly by blocking its protease domain. Our findings shed light on a new aspect of chlamydial host co-evolution which involves the recruitment of host cell proteins into the inclusion to control the activation of bacterial proteases like CPAF that are important for the normal development of *Chlamydia*.

## Introduction

*Chlamydia trachomatis* is an obligate intracellular pathogen, which causes the eye disease trachoma and different sexually transmitted diseases. Due to its intracellular life style and the dependency on host metabolites for replication, *Chlamydia* has evolved numerous unique characteristics that support its own survival without damaging the host cell. One of the fascinating aspects of its life cycle is the establishment of a membrane-bound vesicle called inclusion inside which it completes its biphasic developmental cycle and at the same time utilizes it as a barrier to protect itself from cell autonomous defense mechanism of the host (Fischer and Rudel, 2016; Fischer et al., 2017). These bacteria use type III secretion system to release bacterial proteins into the inclusion membrane and beyond into the host cell cytosol and organelles (Betts-Hampikian and Fields, 2010). On the other side, chlamydial proteins secreted via the bacterial type II secretion system including secreted proteases are kept within the inclusion and away from host cell cytoplasm, although release of such factors into the host cell cytosol has been suggested to occur during the late stage of the developmental cycle (Chen et al., 2010b). One such widely studied chlamydial protease is Chlamydial Protease-Like Activity Factor (CPAF), which has initially been shown to cleave numerous host cell proteins in infected cells (Pirbhai et al., 2006; Chen et al., 2009; Christian et al., 2010; Jorgensen et al., 2011; Snavely et al., 2014; Tang et al., 2015). However, most of these CPAF targets turned out to be cleaved during cell lysis and not in intact cells (Chen et al., 2012). Time specific expression and restricted localization of CPAF inside the inclusion lumen during nearly the whole chlamydial life cycle apparently does not allow cleavage of the host cell targets thus avoiding possible premature lysis of the host cell. CPAF possesses autocatalytic activity and activates itself in a concentration dependent manner *in vitro* (Huang et al., 2008; Paschen et al., 2008). However, initiation of CPAF catalytic activity under very low concentrations as they are found inside the chlamydial inclusion is not completely understood. Similarly, nothing is known about the molecular mechanism that controls CPAF activity within the inclusion lumen.

During a microarray study of host cell transcriptome, we encountered a human peptidase inhibitor called PI15 (also called as p25TI) that was differentially regulated during chlamydial infection. PI15 belongs to the cysteine-rich secretory proteins, antigen 5, and pathogenesis-related 1 proteins (CAP) superfamily and is a weak serine protease inhibitor (Koshikawa et al., 1996) predicted to be a secretory protein (Yamakawa et al., 1998; Kaplan et al., 1999; Gibbs et al., 2008). PI15 is expressed in a variety of human and other animal tissues (Yamakawa et al., 1998; Smith et al., 2001; Takemoto et al., 2006). Here we show that PI15 is recruited into the chlamydial inclusion and plays a crucial role in initial CPAF activation.

## Materials and methods

### Cell lines and *Chlamydia trachomatis* infection

HeLa and 293T cells were grown in RPMI 1640 media and 10% fetal bovine serum (FBS) at 37°C and 5% CO_2_. T-REx-293 cells (Invitrogen) were grown in presence of Tetracycline negative FBS (Gibco-BRL) and desired antibiotics (Zeocin and/or Blasticidin). For induction of PI15 expression in T-REx-293 cells, cells were cultured with 0.2 μg/ml of doxycycline for 2 days. These cells were washed with PBS twice before *Chlamydia* infection. All the cell types were generally infected with *C. trachomatis* (L2) at a multiplicity of infection (MOI) of 1 unless otherwise specified. *Chlamydia* infectious progeny was determined by using EB preparations for a second round of infection and subsequent quantification of the secondary progeny by both immunoblotting and immunostaining techniques (Prusty et al., 2013). For immunostaining-based progeny calculation, total number of inclusions were calculated within a frame and were divided by total number of cells (counted from DAPI staining) to get the average inclusion numbers per cell.

### Immunoblotting

For immunoblotting, cells were lysed in Laemmli sample buffer (100 mM Tris/HCl pH 6.8, 4% SDS, 20% glycerin, 1.5% β-mercaptoethanol, 0.2% bromophenol blue) and resolved by 10–12% sodium dodecyl sulfate polyacrylamide gel electrophoresis (SDS-PAGE). Proteins were transferred to polyvinylidene difluoride (PVDF) membranes (Millipore) and blocked with 5% milk/TBS-T. The membranes were then probed with respective primary antibodies and subsequently with HRP-conjugated secondary antibodies. Proteins were detected by enhanced chemiluminescence (ECL, Amersham) in the linear response range using X-ray films.

### Immunofluorescence microscopy

Cells were seeded in 12-well plates on glass coverslips and were infected with *Chlamydia trachomatis* for desired durations. Cells were washed in PBS and fixed with 4% paraformaldehyde for 30 min. After washing, the cells were permeabilized using 0.2% Triton-X100 in PBS for 30 min and blocked with 10% FCS in PBS for 45 min. Cells were incubated for 1 h with the primary antibody in blocking buffer (2% FCS in PBS), washed three times in PBS, and stained with the corresponding secondary antibody. Draq5 or DAPI was used for staining cell nuclei. After washing three times with PBS, samples were mounted onto slides using Mowiol (Carl Roth, Germany). Samples were analyzed on either a Leica DMR epifluorescence or Leica SPE or Leica SP5 confocal microscope. For live cell imaging, HeLa cells were transfected with a construct expressing mCherry tagged PI15 or an empty vector. 6 h after transfection cells were infected with *C. trachomatis* expressing GFP for another 24 h. Afterwards cells were imaged using Leica SP5 microscope.

### Structured illumination microscopy (SIM)

Cells were washed in PBS and fixed with 4% paraformaldehyde for 15 min. After washing, cells were permeabilized using 0.2% Triton X-100 in PBS for 15 min and were blocked with 2% FCS in PBS for 45 min. Cells were incubated for 1 h with the primary antibody in blocking buffer (2% FCS in PBS), washed three times in PBS, and stained with the corresponding secondary antibody. Cells were then incubated in 1:2000 DAPI solution in 2% FCS-PBS. After washing three times with PBS a post staining fixation process was performed. All samples were imaged on Structured Illumination platform. SIM images were acquired with a Zeiss ELYRA S.1 SR-SIM structured illumination platform. A Plan-APOCHROMAT 63x/1.40 oil objective [lateral resolution (XY): 120 nm, Axial resolution (Z): 300 nm] was used and emission was collected onto a PCO Edge sCMOS camera (effective pixels: 1,280 × 1,280; pixel size 6.5 × 6.5 μm; QE: 57%; dynamic range 15 bit) (Kelheim, Germany).

For GFP/DAPI/Cy5 experiments the 488 nm (100 or 200 mW)/405 nm (50 mW)/642 nm (150 mW) laser lines were used with 488–504 nm emission filters for GFP, 395–405 nm emission filters for DAPI and 645 nm emission filter for Cy5. To prevent spectral cross talk, all SIM data were acquired in alternating excitation mode. SIM reconstruction was performed with the ZEN imaging software (64-bit) with automatic parameters for PSF selection in Structured Illumination mode. For image preparation, the SIM reconstructed images were scaled 2 × 2 with bilinear interpolation then smoothed with a Gaussian blur of pixel radius 0.8. In many cases, for illustration purposes, a max projection in z over the relevant slices was done.

### Quantification of PI15-CPAF co-localization

Pearson's co-localization coefficient (Rr) and signal overlap (R) were determined as described previously (Chowdhury et al., 2017; Fischer et al., 2017). The co-localization analysis was performed on images acquired using a Zeiss ELYRA S.1 SR-SIM structured illumination platform using a Plan-APOCHROMAT 63x oil immersion objective with a numerical aperture of 1.4. The images were reconstructed using the ZEN 2012 image-processing platform with a SIM module. The COLOC2 plugin from FIJI (Bolte and Cordelieres, 2006; Schindelin et al., 2012) was used to determine degree of co-localization and signal overlap (Rr and R). Rr was determined by processing SIM micrographs of 10 different *Chlamydia* infected HeLa cells with ~2 ROI from each picture.

### Antibodies

For immunofluorescence as well as immunoblotting experiments the following antibodies were used. Rabbit monoclonal antibody (ab131209 from Abcam), Rabbit polyclonal antibody (ab113895 from Abcam) against human PI15, mouse monoclonal anti-flag antibody for immunoblotting (F3605, Sigma), mouse monoclonal anti-Flag antibody for immunostaining (F1804, Sigma), mouse monoclonal against human RFX5 (sc-271756, Santa Cruz Biotechnology), mouse monoclonal against human cytokeratin 8 (sc-58737, Santa Cruz Biotechnology), rabbit polyclonal against human vimentin (sc-5565, Santa Cruz Biotechnology), mouse monoclonal against chlamydial Hsp60 (sc-57840, Santa Cruz Biotechnology), mouse monoclonal against human beta-actin (A5441, Sigma). Antibodies against CPAF, cHtrA, IncA were kindly provided by Guangming Zhong and George Haecker. For immunoprecipitation experiments, anti-Flag M2 antibody bound to magnetic beads (Sigma) were used.

### Quantitative real time PCR

For quantitative real time PCR, PerfeCTa qPCR SuperMix (Quanta Biosciences) was used and PCR amplifications were done on a StepOnePlus real time PCR platform (Applied Biosciences) according to the manufacturer's protocol. Data were analyzed using StepOne Software v2.3. The primers used to quantify chlamydial DNA, PI15 mRNA and 5S RNA have been previously described (Prusty et al., 2013).

### Lentivirus mediated gene silencing

HeLa cells with PI15 knockdown were generated by lentivirus-mediated transduction of expression constructs expressing the following 4 shRNAs directed against human PI15 ORF: GGTGCTAAATGAATTGTTATT, CCACATATCCTTGCTATAATT, GAAGATATCGCTCTATTCTTT, CCATGGACCTTCTTACTTATT. Single-cell clones were isolated and cells were cultivated in RPMI-1640 (Gibco-BRL) supplemented with 10% fetal calf serum (FCS).

### Transient silencing of PI15

For siRNA mediated transient silencing of PI15, siRNA pools against human PI15 protein were purchased from SIGMA-Aldrich (MISSION esiRNA human PI15, Cat no. EHU055021-20UG) and Dharmacon (L-013135-00-0005). For transfection of siRNAs, HiPerFect transfection reagent (Qiagen) was used. Control scrambled siRNA (siControl) was purchased from Dharmacon.

### Transient overexpression of PI15

For transient overexpression of PI15, a Myc-DDK-tagged construct was purchased from OriGene (RC213374). mCherry-tagged PI15 expression construct was generated by cloning human PI15 into pmCherry-N1 vector (Clonetch).

### Recombinant CPAF and PI15

Recombinant CPAF constructs were generated by cloning *C. trachomatis* CPAF into either pET28b (C-terminal His-tagged) or pETM11 (N-terminal GST-tagged) constructs. Similarly, human PI15 was cloned into pET28b vector (C- as well as N-terminal His-tagged). TEV protease cut sites are introduced between PI15 ORF and His coding sequences. For recombinant protein expression, protein-coding constructs were transformed into *E. coli* pRARE strain and were grown overnight at 15°C in presence of 0.5 M IPTG. Bacterial cells were pelleted down, lysed with 50 mM Hepes pH = 7.5 containing 100 mM NaCl, 5 mM β-Mercaptoethanol, 0.5 mM PMSF and necessary protease inhibitors. After removal of cell debris, soluble recombinant proteins were eluted by either Glutathione beads or Ni-NTA beads (Thermo Fischer) wherever necessary. Purified His-PI15-His proteins were digested with TEV protease and untagged PI15 proteins were eluted out. Eluted proteins were further purified by gel-filtration chromatography using Supadex 200 and sepharose 6 columns.

### CPAF protease activity assay

For CPAF cleavage activity assay HeLa cell lysate was prepared by lysing HeLa cells in RIPA buffer [50 mM Tris-HCl pH 7.5, 150 mM NaCl, 1% Triton X-100, 1% NP-40, 0.1% SDS, 10% Glycerol and protease inhibitor cocktail (Roche)]. Soluble proteins were separated by centrifugation. Protein concentration was measured by Bradford colorimetric assay. 60 μg of total lysate was used for each assay. For CPAF activity assay, total protein lysates were incubated with different concentrations of CPAF in presence of 1X reaction buffer [25 mM Tris (pH 8.0), 150 mM NaCl, and 3 mM dithiothreitol (DTT)] for 1 h at 30°C. For study of inhibitor activity of PI15, both recombinant CPAF and PI15 were incubated for 30 min at 30°C prior to the addition of cell extract.

## Results

### PI15 expression is differentially regulated during *Chlamydia* infection

**Figure 1:**
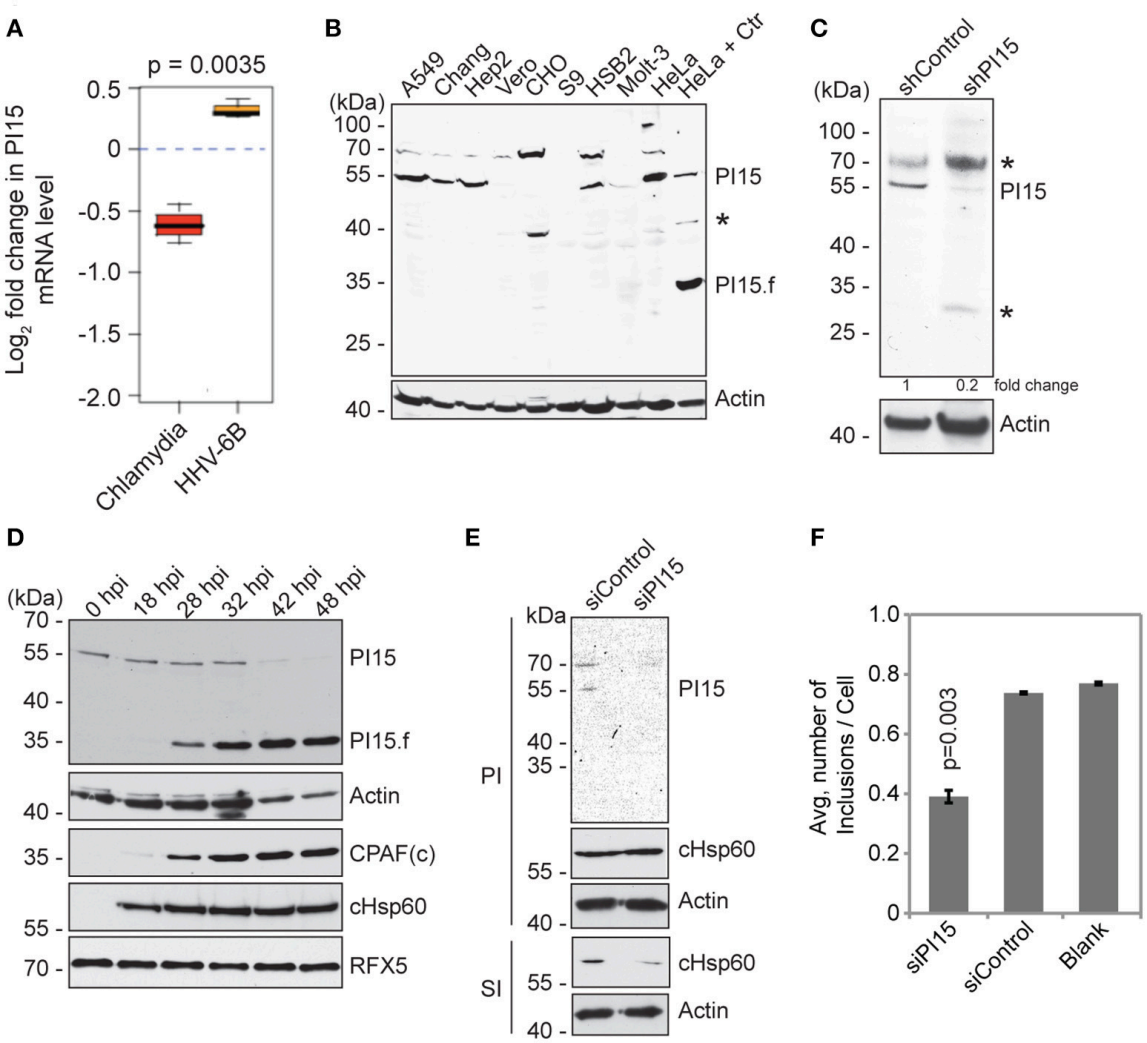
PI15 expression is differentially regulated during *Chlamydia* infection. **(A)** PI15 is differentially regulated at the transcriptional level during *Chlamydia* and HHV-6B infection. HeLa cells were infected with *C. trachomatis* or HHV-6B for 24 h and PI15 RNA was detected by microarray analysis. **(B)** PI15 protein expression was analyzed in different cell lines by immunoblotting. Lysates from HeLa cells infected with *C. trachomatis* for 24 h were compared with the same from uninfected HeLa cells in the last two lanes. A549, human lung cells; Chang, human liver cells; Hep2, human epidermoid cancer cells; Vero, monkey kidney epithelial cells; CHO, Chinese hamster ovarian cells; S9, human bronchial epithelial cells; HSB-2 and Molt-3, human T-cell leukemia cells; HeLa, human cervical epithelial cells. Actin was used as a loading control. ^*^, Unknown protein. **(C)** Identification of PI15 species that is silenced upon PI15-specific shRNA expression. HeLa cells were infected with either control lentiviral particles or with those that express shRNA against PI15. Protein lysates were verified for protein expression by immunoblotting. ^*^, Unknown protein. **(D)** PI15 protein was cleaved during *Chlamydia* infection. HeLa cells were infected with *C. trachomatis* for different time intervals. Total lysates were prepared on ice using Laemmli sample buffer. Samples were processed for immunoblotting. CPAF and cHsp60 expression were tested as a control for chlamydial infection. Actin was used as a loading control and RFX5 expression was tested to monitor potential unwanted post lysis cleavage by CPAF. hpi, hours post infection; PI15.f, possible cleaved fragment of PI15; CPAFc, C-terminal fragment of activated CPAF. **(E)** siRNA-mediated silencing of PI15 inhibits chlamydial progeny formation. HeLa cells were transfected with siRNA against human PI15 for 72 h and were subsequently infected with *C. trachomatis* for another 36 h [Primary infection (PI)]. Parallel sets of experiments were carried out with cells transfected with scrambled siRNA controls. Secondary infection (SI) was carried out by applying lysed primary infected cells to fresh cells to test for *Chlamydia* growth and progeny formation under the influence of siRNAs. **(F)** Average number of chlamydial inclusions per cell was counted during secondary infection. Data represents ± SEM from 3 independent experiments.

We have previously observed decreased chlamydial growth in presence of human herpesvirus 6 (HHV-6) co-infection (Prusty et al., 2012). To identify host factors that might influence chlamydial growth in presence of HHV-6, we performed a microarray based host cell transcriptome analysis and found PI15 to be differentially regulated during *Chlamydia* and HHV-6 infection (Figure 1A). The same result was subsequently verified by RT-PCR analysis (Figure S1A). We therefore investigated the role of PI15 during chlamydial infection. Although PI15 expression was observed in various types of animal tissues by *in situ* hybridization (Smith et al., 2001; Takemoto et al., 2006), the apparent molecular weight of PI15 and its expression dynamics in human tissues is unclear. Hence, we analyzed the expression pattern of PI15 in a wide range of cultured cells by immunoblotting. Instead of the expected mass of ~25 kDa, we detected protein bands at ~55 kDa under various denaturing conditions in majority of cell types including HeLa cells (Figure 1B). As PI15 has a high content of cysteine residues (5% of all amino acids), oligomeric forms of PI15 may form under natural expression conditions by disulfide bond formation. In addition, PI15 possesses a predicted glycosylation site (Yamakawa et al., 1998) and differential glycosylation may contribute to mass differences of the mature protein. Immunoprecipitation (IP) using a monoclonal antibody against PI15 followed by mass spectrometry analysis validated that the observed bands at ~55 kDa were indeed PI15.

Stable knock down of PI15 in HeLa cells using shRNA-based lentiviral vectors confirmed the molecular mass of denatured PI15 identified by immunoblotting to be ~55 kDa (Figure 1C). Interestingly, we detected an additional band of PI15 at ~35 kDa with concurrent loss of the protein bands of higher masses in *Chlamydia* infected HeLa cells (Figure 1B). Hence, we further analyzed expression dynamics of PI15 in HeLa cells during different time points of *Chlamydia* infection (Figure 1D). Corroborating our results, we detected gradual loss of ~55 kDa forms of PI15 at later infection time points with simultaneous appearance of low-molecular PI15 species after midterm of the chlamydial life cycle (Figure 1D), which might represent cleaved PI15 fragments. Previous work has demonstrated that the chlamydial protease CPAF can cause unspecific degradation of proteins upon lysis of the cells, which can be prevented by adjusting the lysis conditions (Rajalingam et al., 2008; Chen et al., 2012). Hence potential artificial cleavage of PI15 was verified by simultaneous detection of the uncleaved CPAF *in vitro* substrate RFX5 (Zhong et al., 2001) that remained intact in lysates collected at different time points post infection. Furthermore, processing of PI15 to a lower molecular weight form depended on the multiplicity of *Chlamydia* infection (MOI) (Figure S1B) and increased with increasing MOI.

We then investigated if changes in expression of PI15 affect chlamydial development. For this, we transiently suppressed PI15 expression using siRNAs in HeLa cells. Upon *Chlamydia* infection in these cells, we neither observe a difference in chlamydial growth nor inclusion size during primary infection in comparison to control siRNA transfected cells (Figure 1E). We then lysed these *Chlamydia* infected cells and added the infectious cell lysates to fresh cells to determine the infectious progeny. These assays revealed decreased numbers of chlamydial progeny in absence of PI15, which was quantified by counting the number of chlamydial inclusions during secondary infection (Figure 1F) and by calculating chlamydial genome equivalents by quantitative PCR (Figure S1C).

The adverse effect of PI15 suppression on chlamydial propagation implicated a positive effect of PI15 on *Chlamydia* infection. Hence we first transiently overexpressed Flag-tagged PI15 in 293T cells (Figure S1D) for 24 h and then infected these cells with *Chlamydia*. Similarly, as previously observed for PI15 silencing, PI15 overexpression did not affect chlamydial primary infection, as chlamydial Hsp60 (cHsp60) protein levels remained unchanged (Figure S1D). However, we observed a decrease in chlamydial progeny as evaluated by immunoblotting (Figure S1E), counting of chlamydial inclusions (Figure S1F), and chlamydial genome copy number calculation (Figure S1G). Taken together, our results suggest a role for PI15 in chlamydial growth whose tight regulation is required for normal chlamydial development.

### PI15 is recruited to the chlamydial inclusion

**Figure 2:**
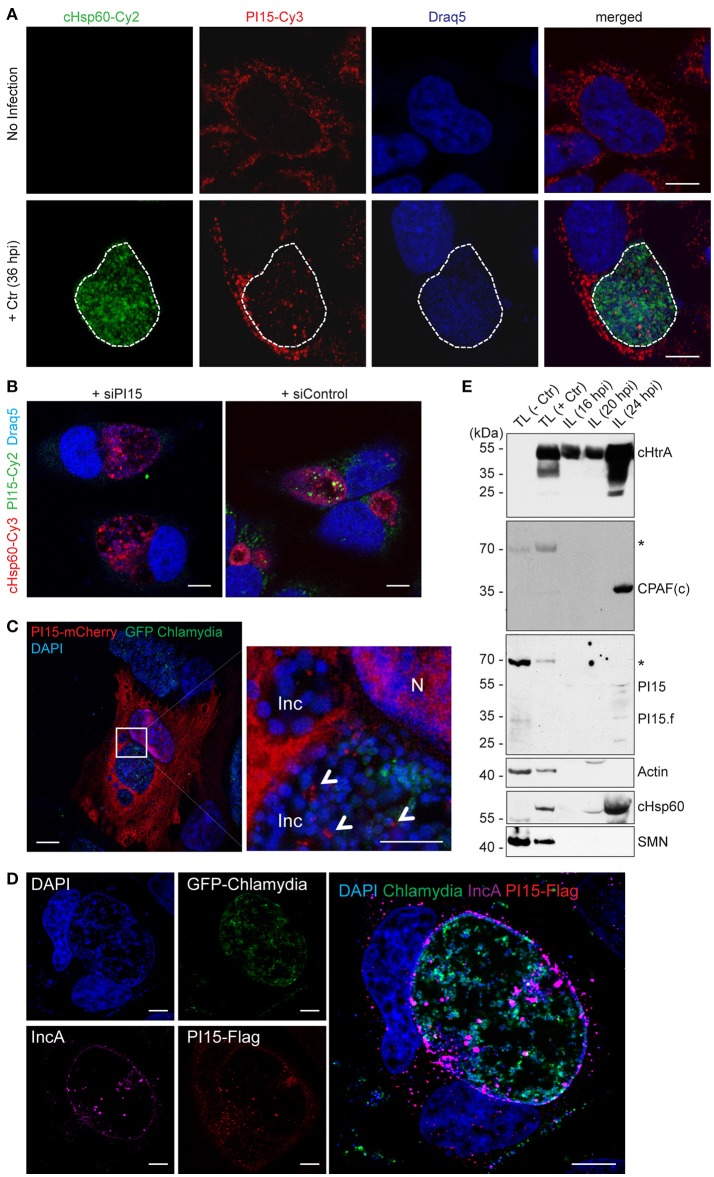
PI15 is recruited into the chlamydial inclusion. **(A)** PI15 is localized within the chlamydial inclusion. HeLa cells were either left uninfected or infected with *C. trachomatis* (Ctr) for 24 h. Cells were immunostained using PI15 or *Chlamydia*-specific Hsp60 (cHsp60) antibodies. Draq5 was used to stain DNA. The chlamydial inclusion is marked with a white dotted line. **(B)** Validation of the PI15 antibody by RNAi. HeLa cells were either transfected with siRNAs against PI15 or control siRNAs. 72 h post transfection, cells were infected with *C. trachomatis*. Cells were immunostained as mentioned above. **(C)** PI15 localizes in the lumen of the chlamydial inclusion. HeLa cells were transiently transfected with a mCherry-tagged PI15 construct. 48 h after transfection, cells were infected with *C. trachomatis* expressing GFP. Cells were fixed 30 h post infection and stained with DAPI. SIM microscopy was used to study the localization of mCherry-PI15 within the chlamydial inclusion. Inc, inclusion; N, Nucleus. **(D)** Recruitment of Flag-tagged PI15 to the chlamydial inclusion. HeLa cells were transiently transfected with a Flag-tagged PI15 construct for 48 h and then infected with *C. trachomatis* expressing GFP. Cells were fixed and immunostained using antibodies against Flag and chlamydial IncA. **(E)** PI15 was detected within purified chlamydial inclusions. HeLa cells were infected with *C. trachomatis* for different time intervals. The chlamydial inclusion was purified from the cells and protein lysates (IL) were prepared from purified inclusions. Total cell lysates (TL) from uninfected and 24 h infected cells were used as control. Actin and survival motor neuron (SMN) were detected as markers for cytosol and nuclei to evaluate inclusion purity. hpi, hours post infection. ^*^, non-specific band. Scale bars in all panels, 10 μM.

As a subsequent step, the subcellular localization of PI15 was investigated by confocal immunofluorescence microscopy using monoclonal antibodies against PI15. PI15 was detected in vesicular structures in the cytosol of the infected cell but also inside the chlamydial inclusion (Figure 2A). Numerous different control experiments were performed to support the localization of PI15 within the inclusion. RNAi-mediated knockdown of PI15 prevented any staining with PI15-specific antibodies validating the specificity of the PI15 antibody (Figure 2B; for individual channel images see Figure S2A). In a separate set of experiments, we transiently over-expressed N-terminal mCherry-tagged PI15 in HeLa cells and infected these cells with *C. trachomatis* that constitutively express GFP. Cells were fixed and stained with DAPI without using any antibody and were analyzed by structured illumination microscopy (SIM). We detected mCherry-tagged PI15 within the lumen of the chlamydial inclusion (Figure 2C). In cells expressing mCherry alone without fusion to PI15, mCherry was not detected in the inclusion lumen when infected with GFP-expressing *Chlamydia*. Furthermore, we transiently over-expressed Flag-tagged PI15 in HeLa cells and infected these cells with GFP-expressing *Chlamydia*. Immunostaining with a flag-specific antibody corroborated previous results showing intra-inclusion localization of PI15 (Figure 2D). Co-staining with IncA antibody validated localization of PI15 within inclusion lumen. Recent studies indicated that host cell proteins are released from the cytosol into the chlamydial inclusion as a consequence of chemical fixation of specimen (Kokes and Valdivia, 2015). To exclude any fixation artifacts, we carried out live cell imaging using mCherry-tagged PI15 and GFP-expressing *Chlamydia* (Figure S2B) and detected mCherry-PI15 within the chlamydial inclusion lumen. In cells transfected with the empty vector control, mCherry proteins were not detected within the inclusion lumen (Figure S2C). These results further corroborated the presence of PI15 within the chlamydial inclusion lumen. In a last set of experiments, we developed an inclusion purification technique (Herweg et al., 2015) and purified *C. trachomatis* inclusions at 3 different time points post infection. Inclusions were extremely fragile after 24 h post infection and were hence not included in the study. ~55 kDa as well as low molecular weight forms of PI15 was detected in purified chlamydial inclusions by immunoblotting (Figure 2E). Purity of isolated inclusions was verified by detection of cytoplasmic as well as nuclear (actin, SMN) proteins, which did not co-purify with the inclusions.

To test if PI15 is also present in the inclusion of other serovars, we performed immunofluorescence studies in cells infected with *C. trachomatis* serovar D. We did not see any difference in localization of PI15 within chlamydial inclusions of serovar D in comparison to serovar L2 (Figure S2D). Thus our results demonstrate that PI15 is recruited into the chlamydial inclusion and localizes within the inclusion lumen.

### PI15 partially co-localizes with CPAF within the inclusion lumen

**Figure 3:**
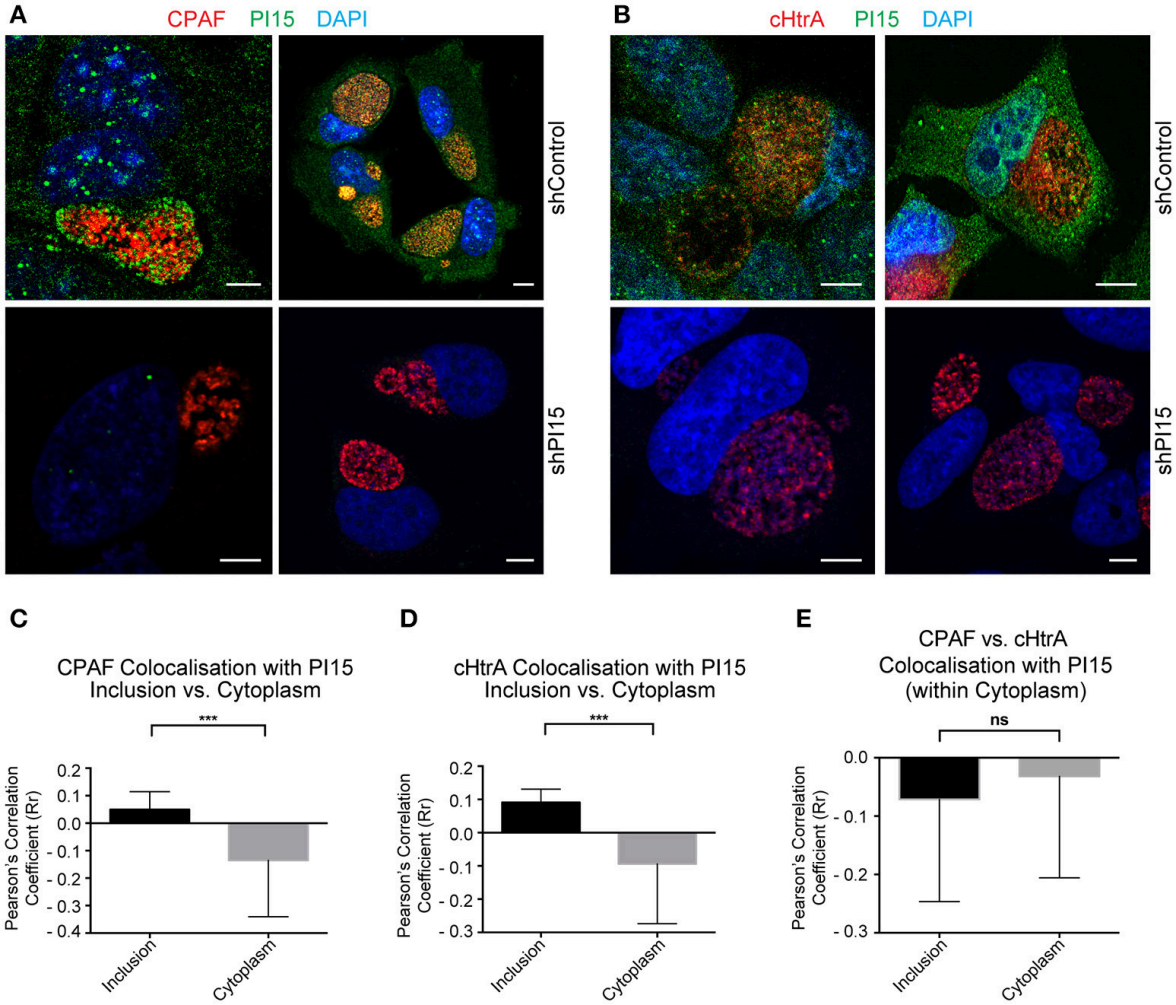
PI15 partially co-localizes with CPAF. **(A)** Confocal microscopy of HeLa cells showing partial PI15 and CPAF co-localization within inclusion lumen. HeLa cells transduced with control (upper panel) or shPI15-expressing lentiviral vectors (lower panel) were infected with *C. trachomatis* for 30 h and then immunostained using antibodies against PI15 and CPAF. DAPI was used to stain DNA. Two representative images are shown from different biological replicates. **(B)** Chlamydial cHtrA and PI15 co-localization was studied under similar infection conditions as in **(A)**. **(C)** Quantitative analysis of PI15 and CPAF co-localization within chlamydial inclusions and host cell cytoplasm. Confocal images of a *Chlamydia* infected HeLa cell immunostained as above were used for quantitative analysis. Pearson's overlap coefficient (Rr; representing the degree of overlap between two groups of particles in the image) were obtained by processing confocal images of 38 different *Chlamydia* infected HeLa cells with ~2 ROI from each picture. **(D)** Similar analysis was carried out for chlamydial cHtrA. **(E)** CPAF and cHtrA co-localization with PI15 within the host cell cytoplasm was compared as above. ^***^ ≤ 0.0005. Scale bars in all panels, 10 μM.

PI15 has been described as a serine protease inhibitor (Koshikawa et al., 1996). Since *Chlamydia* secretes several serine proteases, including CPAF (Huang et al., 2008) and cHtrA (Wu et al., 2011) we asked if PI15 associates with those proteases inside the inclusion. Immunofluorescence studies using antibodies against CPAF and PI15 revealed a partial co-localization of PI15 with CPAF aggregates within the chlamydial inclusion lumen (Figure 3A). We also detected similar co-localization of PI15 with cHtrA, (Figure 3B). We determined the degree of co-localization of CPAF and PI15 (Figure 3C) as well as cHtrA and PI15 (Figure 3D), which revealed partial but positive correlation for both chlamydial proteases and PI15. We did neither detect CPAF nor cHtrA in host cell cytoplasm resulting in a negative correlation between these two proteins and PI15 in the cytosol (Figure 3E). Transverse and coronal cut (Figure S3A) z-stack analysis of CPAF-PI15 co-localization further confirmed these findings (see Supplementary Video Files 1–4).

### CPAF is specifically localized within chlamydial inclusions

CPAF has been detected within host cell cytoplasm under various fixation conditions (Yang et al., 2015). However, we could not detect cytoplasmic CPAF under the cell culture and fixation conditions we used. Hence we compared our fixing conditions to that of others and found that minor changes in the fixation procedure caused major differences in the localization of CPAF either in or outside the inclusion. Fixation of *Chlamydia*-infected cells with ice cold methanol for 10 min was highly effective in preventing leakage of CPAF into the host cell cytoplasm. As methanol fixing inhibits cHtrA antibody binding, we preferred to use paraformaldehyde (PFA) at 37°C for the entire work. Interestingly, with 20 min of cell fixation with 4% PFA at 37°C, we could not detect CPAF in the host cell cytoplasm whereas the same fixation solution and time (4% PFA for 20 min) used at room temperature instead of 37°C resulted in cytoplasmic CPAF in almost every infected cell (Figure S3B) leading to false positive correlation between cytoplasmic CPAF and PI15 (Figure S3C). However under both fixing conditions, we did not detect cytoplasmic cHtrA (Figure S3B) suggesting that the secreted proteases cHtrA and CPAF behave differently in the inclusion. cHtrA appears to remain bound inside the inclusion whereas CPAF seems to be present in a soluble form within inclusion lumen, which is released into the cytosol if the inclusion membrane gets leaky. Infecting cells with the CPAF negative RST17 chlamydial strain, obtained by chemical mutagenesis that contains a nonsense mutation in the CPAF gene *cpa* (CTL0233) (Snavely et al., 2014), confirmed CPAF-specific staining (Figure S3D). Chlamydial CPAF is not secreted into inclusion lumen under persistent bacterial infections (Wang et al., 2011). We created such persistent chlamydial infection by co-infecting *Chlamydia*-infected cells with HHV-6A (Prusty et al., 2012). Under these conditions, we observed RB-bound CPAF within the inclusion lumen (Figure S4A), which also co-localized with PI15 (Figures S4B–S4H). These results suggest that PI15 has a strong binding affinity to CPAF and can be co-localized with CPAF under various experimental conditions.

### PI15 forms a functional complex with CPAF

**Figure 4:**
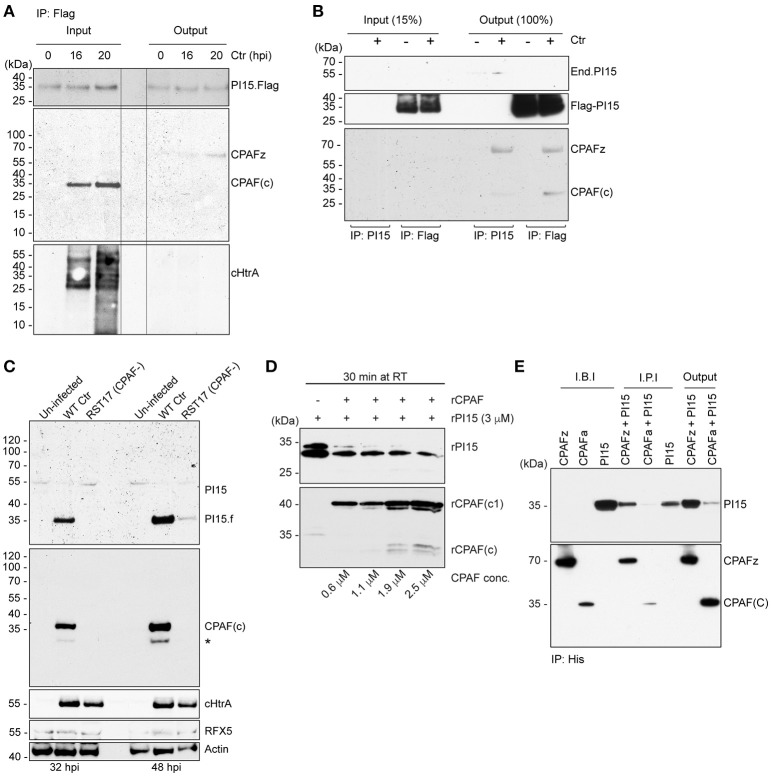
PI15 interacts with the CPAF zymogen. **(A)** PI15 binds to the CPAF zymogen. Co-immunoprecipitation (Co-IP) experiments were carried out using HeLa cells that were transiently transfected with a PI15-Flag constructs. Cells were infected with *C. trachomatis* for different time intervals. Total cellular lysates were prepared and used for Co-IP using Flag beads. **(B)** PI15 interacts with CPAF. Co-IP experiments were carried out using T-REx-293 cells that inducibly express PI15-Flag. Cells were induced with 0.1 μg/ml of AHT for 48 h and then infected with *C. trachomatis* for another 24 h. Total cellular lysates were prepared and used for Co-IP using either commercial Flag beads or agarose beads with covalently cross-linked PI15 antibodies. **(C)** Degradation of PI15 depends on CPAF. HeLa cells were infected with *Chlamydia* wild type or the *Chlamydia* CPAF mutant RST17 (CPAF^−^), for 32 or 48 h, respectively. PI15 as well as CPAF expression was analyzed by immunoblotting. Post lysis degradation of substrates by CPAF was monitored by testing RFX5 expression. ^*^, PI15 bands from the previous blot. **(D)** CPAF cleaves PI15 *in vitro*. Recombinant PI15 was incubated in the presence of increasing concentrations of recombinant CPAF at room temperature for 30 min and were analyzed by immunoblotting. **(E)** CPAF interacts with PI15 *in vitro*. Co-IP was carried out using an inactive full-length recombinant CPAF or active CPAF (both His-tagged) and recombinant PI15 (without any tag). I.B.I., Input samples before incubation; I.P.I., Input after 2 h incubation at room temperature; CPAFz, CPAF zymogen; CPAFa, CPAF active; CPAF(c), c-terminal fragment of active CPAF; CPAF(c1), intermediate c-terminal fragment of active CPAF; End.PI15, endogenous PI15.

Partial co-localization of PI15 and CPAF suggested that both these proteins might be involved in a functional complex within the chlamydial inclusion lumen. CPAF is translated as an inactive zymogen, which during secretion into the inclusion lumen is N-terminally processed by a signal peptidase. The full-size zymogen without signal peptide dimerizes in the inclusion lumen and is then further processed into two fragments to form the active protease complex (Zhong et al., 2001; Huang et al., 2008; Chen et al., 2009). Immunoprecipitation of Flag-tagged PI15 in cells transiently expressing Flag-PI15 using anti-Flag antibodies pulled down the inactive CPAF zymogen (Figure 4A) during early stages of *Chlamydia* infection. However, we did not detect active and processed CPAF co-immunoprecipitating with PI15. Similarly, we did not detect cHtrA co-immunoprecipitating with PI15. We repeated co-immunoprecipitation experiments using T-REx-293 cells with an anhydrous tetracycline (AHT)-inducible Flag-PI15 expression construct. Also in these cells, the CPAF zymogen co-immunoprecipitated with Flag-PI15. However, occasionally we also detected active CPAF co-immunoprecipitating with overexpressed Flag-PI15 (Figure 4B, Figure S5A). We previously observed the loss of the ~55 kDa PI15 isoform during late stages of *Chlamydia* infection (Figure 1D). Interestingly, in cells infected with RST17 (CPAF^−^) the high-molecular mass forms of PI15 remained uncleaved whereas the low-molecular PI15 forms were strongly reduced in comparison to wild type *Chlamydia* infected cells (Figure 4C, Figure S5B), suggesting that CPAF mediates this conversion during *Chlamydia* infection. To validate CPAF-mediated PI15 cleavage, we expressed recombinant CPAF and PI15 (rPI15) in *E. coli*. During preparation of rPI15, we observed self-cleavage of PI15 leading to the formation of two different sized PI15 products (Figure S6). It is noteworthy that an N-terminal cleavage site for PI15 has been predicted previously (Yamakawa et al., 1998). *In vitro* CPAF protease activity assays, using active recombinant CPAF and rPI15 proteins, were carried out where both the proteins were incubated with each other in presence of a suitable reaction buffer. Results showed cleavage of rPI15 by CPAF (Figure 4D). To further investigate the interaction of CPAF with PI15, we created a recombinant His-tagged CPAF protein containing the complete N-terminal sequence that prevents auto processing of CPAF (Chen et al., 2010a,b). Co-IP of this inactive full-length CPAF using Ni-NTA beads pulled down PI15 protein complexes efficiently (Figure 4E) whereas active His-CPAF cleaved most of the recombinant PI15 proteins. Based on these results we argue that the excess PI15 in induced T-REx-293 cells inhibited CPAF protease activity that is required for the release of PI15 from the PI15-CPAF complex. This explains why processed CPAF forms were occasionally detected together with PI15 under these conditions. In conclusion, these results support a model in which the CPAF zymogen forms a complex with PI15. Once activated, CPAF cleaves PI15 releasing the active CPAF from the complex.

### PI15 expression modulates CPAF maturation

**Figure 5:**
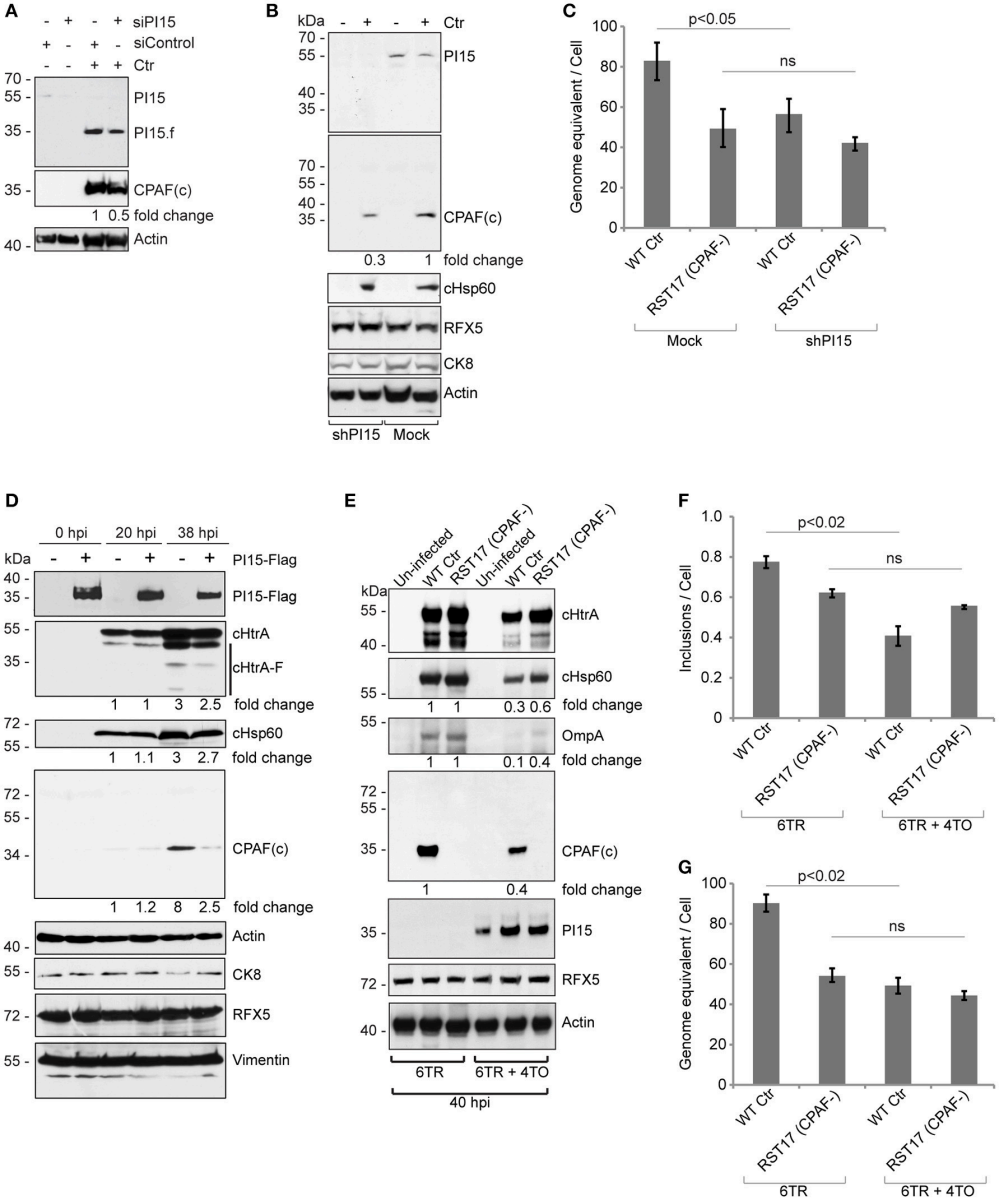
PI15 silencing and over-expression inhibits CPAF activation. **(A)** Cells transfected with control (siControl) or PI15 (siPI15) siRNAs were infected with *Chlamydia* and CPAF maturation was accessed by immunoblotting. Total lysates were prepared from both siPI15 as well as siControl transfected cells after 24 h of *Chlamydia* infection. **(B)** Effect of silencing of PI15 on CPAF maturation was accessed in HeLa cells with lentivirus-mediated stable knock down of PI15. Lysates of cells were harvested 24 h post infection when PI15.f cannot be detected. Cells with empty vector control were tested in parallel. kDa, molecular weight of protein markers represented as kilodaltons. **(C)** Progeny formation of *Chlamydia* is affected by suppression of PI15 expression. HeLa cells with normal PI15 expression and with shRNA-mediated knock down PI15 were infected with wild type *Chlamydia* (Wt Ctr) or the RST17 (CPAF-) mutant. Cells were lysed 36 h post infection and equal amounts of cell lysates were used to infect fresh cells. Chlamydial DNA was quantified 24 h post infection by qPCR and was normalized to the total cell count. Data represent ± SEM from 3 independent experiments. **(D)** Transient over-expression of PI15 inhibits CPAF maturation. 293T cells were transiently transfected with Flag-tagged PI15 for 48 h and were then infected with *C. trachomatis* for different time intervals. Total lysates were prepared and processed for immunoblotting. CK8, cytokeratin-8; −, Empty vector; +, PI15-flag. Quantities of chlamydial proteins (cHtrA full length form, cHsp60 and CPAF) were determined by normalization to actin. **(E)** Similar experiments were carried out in T-REx-293 cells carrying a Doxycycline-inducible gene of PI15-flag under 4TO vector backbone. Repressor protein is expressed under the 6TR vector backbone. Cells carrying 6TR alone were used as control. T-REx-293 cells were induced for 48 h with Doxycycline and then washed thoroughly with PBS before *Chlamydia* infection. hpi, hours post infection; −, Empty vector; +, PI15-flag. **(F, G)** Quantification of chlamydial growth in presence of PI15. Secondary infection assays were carried out from the above experiment, to determine effects of stable PI15 overexpression on chlamydial development. Lysed infected cells were used to infect fresh cells and the number of chlamydial inclusions was counted and presented as bar diagram **(F)**. Chlamydial DNA was quantified 24 h post infection by qPCR and was normalized to the total cell count **(G)**. Data represent ± SEM from 3 independent experiments.

To understand the functional significance of the CPAF-PI15 interaction, we analyzed CPAF expression and maturation under RNAi-mediated PI15 suppression. We observed decreased CPAF activation if PI15 levels were reduced (Figure 5A). Stable knockdown of PI15 in HeLa cells also caused a similar decrease in processed CPAF during *Chlamydia* infection (Figure 5B; see lane 2 vs. 4). To understand the link between PI15 and CPAF activation and the impact on chlamydial growth, we compared the progeny formation between wild type and RST17 (CPAF^−^) *Chlamydia* in presence or absence of PI15 (Figure 5C). We observed a significant decrease in the progeny of wild type *Chlamydia* upon PI15 suppression (Figure 5C). However, progeny formation of RST17 (CPAF^−^) *Chlamydia* was not significantly affected in PI15-depleted cells (Figure 5C) suggesting that PI15 functionally interacts with CPAF in infected cells.

To further study a possible functional association between PI15 and CPAF, we transiently over-expressed Flag-tagged PI15 in 293T cells. Overexpression of PI15 decreased the amount of active CPAF 36 h post infection (hpi) but had no effect at 20 hpi when only low levels of CPAF were detected (Figure 5D). A similar downregulation of processed cHtrA was not detected in these experiments (Figure 5D). Post lysis degradation artifacts by chlamydial proteases were excluded by testing the blots for the *in vitro* CPAF substrates Vimentin, RFX5 and CK8 (Zhong et al., 2001; Snavely et al., 2014). In order to test a homogeneous cell population expressing PI15, we used previously described inducible T-REx-293 cells that stably express Flag-PI15 under the control of an inducible promoter. Using these cells, we compared the inhibitory effects of PI15 on wild type *Chlamydia* and the RST17 (CPAF^−^) mutant (Figure 5E). Induction of PI15 expression inhibited growth of wild type *Chlamydia*, however the inhibitory effect on RST17 (CPAF^−^) was not significant. Quantification of chlamydial progeny in secondary infection assays confirmed the inhibition of the development of wild type *Chlamydia* but not of RST17 (CPAF^−^) upon PI15 overexpression (Figures 5F,G). Thus our results suggest that both absence of PI15 as well as overexpression of PI15 have an impact on the activation of CPAF, which in turn regulate chlamydial progeny formation.

### PI15 functions as an activator and inhibitor of CPAF

**Figure 6:**
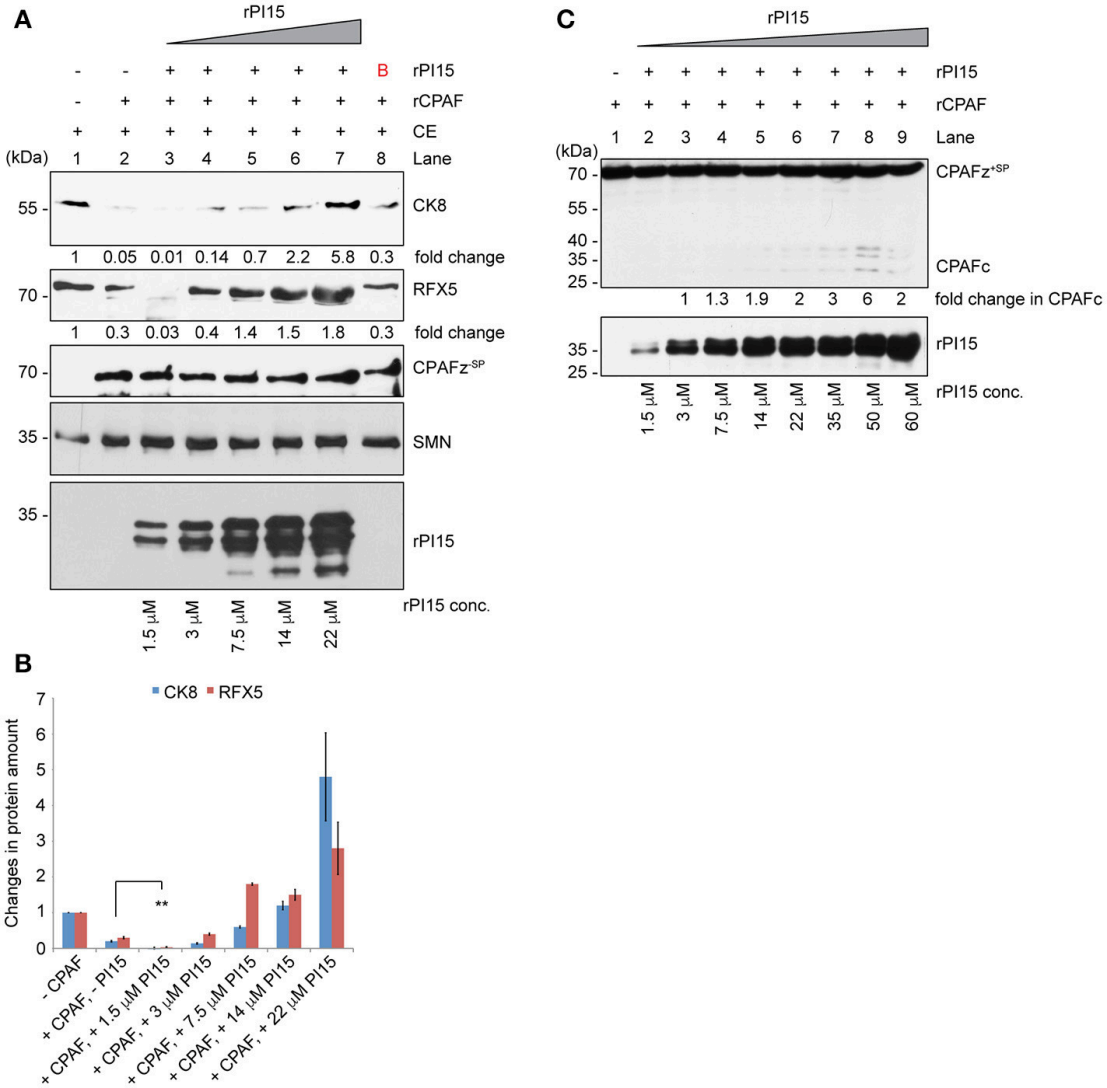
PI15 supports initial CPAF activation. **(A)** Effect of PI15 on CPAF protease activity was studied *in vitro*. 60 μg of total cell extract (CE) from HeLa cells were incubated with 1 μM recombinant CPAF zymogen lacking the signal peptide (CPAFz^−SP^) for 30 min at room temperature in absence or presence of increasing concentrations of recombinant PI15 (rPI15). CPAF protease activity was measured by detecting the CPAF substrates Cytokeratin-8 (CK8) and RFX5 by immunoblotting. SMN and actin were tested as controls as they are not affected by CPAF protease activity and therefore served as loading controls. **(B)** Unrelated protein. **(B)** Both CK8 and *RFX-5* protein bands were quantified from the above blot. Quantification results from 3 independent experiments were plotted as a bar graph. ^**^ ≤ 0.005 **(C)** PI15 supports the initiation of CPAF activation. Recombinant CPAF zymogen carrying the signal peptide sequences (CPAFz^+SP^) was incubated with different concentrations of recombinant PI15 (rPI15) for 30 min at room temperature. CPAF and PI15 were detected by immunoblotting.

To test the functional significance of a PI15-CPAF zymogen complex, we performed *in vitro* CPAF protease activity assays using a fixed concentration of rCPAF zymogen lacking N-terminal signal peptide sequence (CPAFz^−SP^) and variable amounts of rPI15 in presence of 60 μg of total HeLa lysates. Interestingly, we detected enhanced CPAF protease activity in the presence of very low concentrations of rPI15. However, increasing concentrations of rPI15 inhibited CPAF protease activity (Figures 6A,B). To investigate if PI15 supports CPAF activation, we utilized a full-length CPAF zymogen together with the N-terminal signal peptide (CPAFz^+SP^) for further experimentation. CPAFz^+SP^ in contrast to the CPAF zymogen lacking the N-terminal signal sequence (CPAFz^−SP^) fails to activate itself by autoprocessing when incubated at experimental conditions and can therefore be used for PI15 activation studies. Interestingly, we detected processed CPAFz^+SP^ in presence of increasing concentrations of PI15 (Figure 6C), indicating that PI15 induces the processing of CPAFz^+SP^. Our results suggest that the oligomerizing properties of PI15 may position CPAF zymogens in close proximity thereby initiating the auto-processing activity of CPAF. This assumption is also in agreement with the observation that high concentrations of PI15 block proteolytic cleavage sites within CPAF and prevent its substrate processing.

## Discussion

Chlamydial CPAF is a highly active serine protease that is expressed from the mid-term to the end of the chlamydial developmental cycle. Extensive biochemical studies have demonstrated the auto-processing of CPAF in a concentration dependent manner (Huang et al., 2008). However, CPAF is expressed at a very low concentration during the mid-phase of the chlamydial life cycle, which may not suffice to initiate its auto-processing activity. In addition, previous results demonstrated that CPAF is functionally active at early infection time points (Christian et al., 2010; Jorgensen et al., 2011) when CPAF concentrations are low and auto-processing may not occur. We suggest here that CPAF utilizes a host cell protein PI15 to initiate its auto-processing during the early stages of infection since silencing of PI15 caused decreased levels of mature CPAF in infected cells (Figures 5A,B). We also show inhibitory effects of increased concentrations of PI15 on the CPAF protease activity. This inhibitory effect of PI15 is counteracted by cleavage of PI15 possibly within the inclusion lumen. Our study thus provides an example of a host cell CPAF substrate, which is localized within the chlamydial inclusion.

We observed a partial co-localization of PI15 with chlamydial CPAF within the inclusion lumen. Analysis of the super resolution images revealed that PI15 co-localizes with CPAF only inside the inclusion lumen. Since CPAF was only detected in the inclusion lumen, a whole cell co-localization analysis for PI15-CPAF interactions could not be performed. Instead, we selected entire chlamydial inclusions as regions of interests (ROIs) and performed co-localization analysis on these areas. Pearson's co-localization coefficient (Rr) indicated a correlation between the localization of the PI15 with the CPAF within the inclusion lumen. On an individual basis, the PI15 particles which do co-localize with CPAF exhibited ~80% signal overlap (R) within the inclusion lumen indicating an interaction between the two proteins.

The mechanism by which PI15 activates CPAF requires further investigation. We observed consistently high-molecular complexes of PI15, which indicates that this protein either has a stable conformation as a multimer or is post-translationally processed to generate higher molecular weight isoforms. Intriguingly, these high-molecular weight forms were detected under full denaturing and reducing conditions. A small number of proteins including ATP synthase retain its oligomeric forms even under harsh denaturing conditions. In addition, other features including hydrophobic interactions and covalent dityrosine bridging (Atwood et al., 2004) are known to affect complex formation of proteins under experimental conditions. This aggregation behavior of PI15 may be instrumental for the activation of CPAF since high-molecular weight complexes disappeared in the course of chlamydial growth upon CPAF activation,

Recruitment of host cell proteins into chlamydial inclusion is not well studied. Being an intracellular obligate pathogen, *Chlamydia* acquires various metabolites from host cells for the completion of its life cycle. Golgi-derived sphingolipids are trafficked into the chlamydial inclusion (Hackstadt et al., 1995). Golgi fragmentation seems to be an essential event during the chlamydial life cycle required to provide lipids to build the chlamydial inclusion (Heuer et al., 2009). PI15 is processed in the Golgi to its mature secreted form (Yamakawa et al., 1998). Hence it is possible that PI15 is recruited into the chlamydial inclusion via Golgi-associated vesicles.

**Figure 7:**
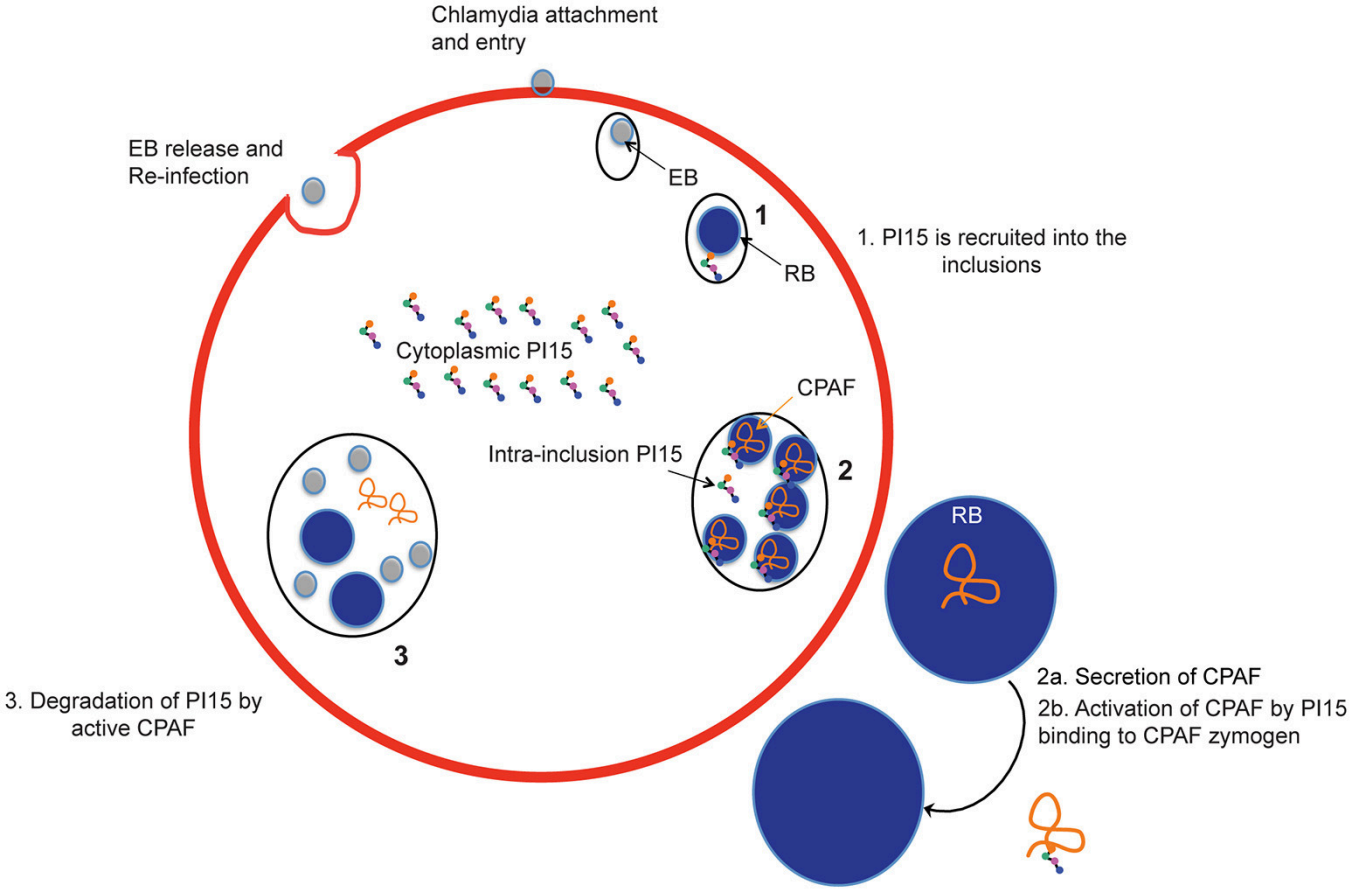
Schematic representation of recruitment of PI15 into inclusions during the chlamydial life cycle. EB, elementary bodies; RB, reticulate bodies.

CPAF is a very active protease and is retained within the inclusion during extended parts of the chlamydial life cycle. CPAF has been shown to play a role in maintaining the integrity of the inclusion and promote virulence (Jorgensen et al., 2011). Our biochemical assays demonstrate an inhibitory effect of high PI15 concentrations on CPAF protease activity. In this line, over-expression of PI15 partially delayed chlamydial growth supporting a functional role for CPAF during the chlamydial life cycle. This is consistent with the observation that CPAF-deficient mutants generated through reverse genetic approaches are impaired in the generation of infectious EBs (Snavely et al., 2014), supporting a key role for CPAF during the second half of the *C. trachomatis* life cycle. Interference with PI15 expression (both silencing as well as over-expression) affected chlamydial progeny formation supporting an important role for CPAF within the inclusion lumen during extended parts of the chlamydial life cycle (see Figure 7). In our study, growth of wild type and CPAF-mutant *Chlamydia* was not similarly affected in the absence of PI15. Inactivation of CPAF alone already reduces chlamydial growth as was shown for RST17 (CPAF^−^) (Snavely et al., 2014). However, in the absence of PI15, CPAF is still activated but clearly less effective. We speculate that CPAF activation at low PI15 levels depends entirely on its concentration-dependent auto-processing activity which may be effective only at late stages of chlamydial developmental. This potentially reduces the overall supportive effects of CPAF on chlamydial growth to an extend as observed in RST17 (CPAF^−^). The exact role of CPAF in chlamydial growth is still not fully understood. We argue that CPAF protease activity is not only required for chlamydial growth as shown by others but also possibly for efficient maturation of infectious EBs. Lack of sufficient functionally active CPAF therefore may delay late chlamydial stages including infectious EB formation.

CPAF was initially characterized as a protease secreted into the cytosol of infected cells during the mid-to-late stage of chlamydial development (Shaw et al., 2002; Dong et al., 2004; Chen et al., 2009). Numerous cellular substrates have been identified and have been attributed to cellular phenotypes mediated by *Chlamydia* infection. However, most of the published cellular CPAF substrates have been shown to be post lysis artifacts (Chen et al., 2012) and the cellular phenotypes previously connected to CPAF are unchanged in cells infected with a CPAF-deficient mutant (Snavely et al., 2014). Whether or not CPAF detected in the host cells cytosol plays any role in *Chlamydia* infection is still a matter of debate (Bavoil and Byrne, 2014). We could not detect CPAF in the cytosol of infected cells until very late in the infection cycle directly before cell lysis, a time point when content of the inclusion may leak out into the host cell cytosol due to an increased permeability of the inclusion membrane and the starting disintegration of the cell.

In conclusion, we show evidence for recruitment of the host cell protease inhibitor protein PI15 into chlamydial inclusion, which might play a major role in controlling the initial activation of the zymogen and its further protease activity essential for chlamydial development.

## Author contributions

BP and TR designed the experiments, BP, NG, and SC performed the experiments, SC performed high-resolution microscopy and quantified PI15 interactions, BP and TR analyzed the data, BP and TR wrote the manuscript.

### Conflict of interest statement

The authors declare that the research was conducted in the absence of any commercial or financial relationships that could be construed as a potential conflict of interest.

## Supplementary material

Figure S1 PI15 regulation and its effect on chlamydial growth.**(A)** PI15 is differentially regulated at transcriptional level during *Chlamydia* and /or HHV-6A infection. HeLa cells were infected with either *C. trachomatis* or *HHV-6A* or both for 24 h. Total RNA was extracted and was used for quantitative reverse transcriptase PCR to analyze PI15 mRNA level. hpi, hours post infection. Data represents ± SEM from 3 independent experiments. ^*^ ≤ 0.05. Ctr, Chlamydia; NI, non-infected control. **(B)** PI15 cleavage was directly correlated with chlamydial load. HeLa cells were infected with *C. trachomatis* for 36 h with different MOI of infection. PI15 expression was studied by immunostaining. **(C)** siRNA-mediated silencing of PI15 inhibits chlamydial progeny formation as quantified by qPCR. HeLa cells were transfected with siRNA against human PI15 for 72 h and were subsequently infected with *C. trachomatis* for another 36 h. Scrambled siRNA were transfected as control. Secondary infection was carried out by applying lysed primary infected cells to fresh HeLa cells to determine *Chlamydia* growth and progeny formation upon PI15 silencing. Total cellular DNA was extracted from infected HeLa cells 36 hpi and was quantified by qPCR. Chlamydial genome equivalent was normalized with cellular genome equivalent to finally calculate genome equivalents per cell. Data represent ± SEM from 3 independent experiments. **(D–G)** Transient over-expression of PI15 inhibits chlamydial growth. **(D)** Flag-tagged PI15 was transiently over-expressed in 293T cells for 24 h. Empty vector (EV) transfection was used as a control. 24 h post transfection, cells were infected with *Chlamydia* for another 24 h. Immunoblotting was carried out to validate PI15 over-expression. cHsp60 was tested to compare chlamydial growth during primary infection (PI). ^*^, Unknown protein. **(E)** Secondary infectivity assay were carried out to determine infectious progeny formation. cHsp60 levels were compared in progeny infected cells **(F)**. Average number of chlamydial inclusions per cell was determined during secondary infection by counting DAPI stained host cell nuclei and inclusions. Data represents ± SEM from 3 independent experiments. ^*^ ≤ 0.05. **(G)** Total cellular DNA was extracted from infected progeny cells 36 hpi and chlamydial genomes were quantified by qPCR as mentioned in **(C)**. Chlamydial genome equivalent was normalized to cellular genome equivalent to finally calculate genome equivalents per cell. Data represent ± SEM from 3 independent experiments.

Figure S2 PI15 localizes within the chlamydial inclusion lumen. **(A)** Validation of PI15-specific antibody staining by RNAi. HeLa cells were either transfected with siRNAs against PI15 or control siRNAs. 72 h post transfection, cells were infected with *C. trachomatis*. Cells were immunostained using PI15 or *Chlamydia*-specific Hsp60 (cHsp60) antibodies. Draq5 was used to stain DNA. Individual channel images are presented. **(B,C)** Live cell imaging was carried out in HeLa cells transiently transfected with mCherry-PI15 **(B)** or an empty mCherry vector **(C)**. 6 h post transfection cells were infected with GFP-expressing *Chlamydia* for another 24 h. Cellular localization of mCherry protein was carried out in live cells using a Leica SP5 microscope. **(D)** PI15 is localized within the inclusion during *C. trachomatis* serovar D infection. HeLa cells were infected with *C. trachomatis* serovar D for 24 h. Cells were immunostained using PI15 or CPAF antibodies. Draq5 was used to stain DNA. Scale bars in all panels, 10 μM.

Figure S3 Comparison of intra-cellular CPAF localization by different fixation methods. **(A)** Schematic diagram showing different planes of sectioning of images used for the study of PI15-CPAF co-localization. **(B)** Confocal images from CPAF or cHtrA stained *Chlamydia* infected cells fixed with PFA either at room temperature (RT) or at 37°C. DAPI staining was used to stain DNA. Inclusions are marked with white dotted lines. **(C)** Cytoplasmic co-localization of CPAF or cHtrA with PI15 was quantified and Pearson's co-efficient was plotted as explained before. ^**^ ≤ 0.005. **(D)** CPAF specific staining was verified by infecting HeLa cells with a mutant strain of *Chlamydia* that does not express CPAF and by using immunostaining. Scale bars in all panels, 10 μM.

Figure S4 CQuantitative analysis of PI15 and CPAF co-localization within inclusions of persistent *Chlamydia*. **(A)** Confocal images showing CPAF and PI15 co-localization within two representative persistent chlamydial inclusions infected with wild type Chlamydia (WT Ctr) and HHV-6A. **(B)** Structured illumination micrograph of a *Chlamydia* infected HeLa cell immunostained for CPAF (green) and PI15 (red). The nucleus was stained with DAPI (blue). **(C)** Co-localisation Mask (coloc mask) was generated using the COLOC2 plugin from FIJI. The white regions illustrate the areas of overlaps between CPAF and PI15 observed only within the chlamydial inclusion. **(D)** The inset from **(A)** was used to generate a signal overlap graph **(E)** by plotting arbitrary fluorescence values against distance traversed by the white line in **(C)**. **(F)** Overlap values (percentage of signal overlap between two particles) and **(G)** Pearson's overlap coefficient (Rr; representing the degree of overlap between two groups of particles in the image) were obtained by processing Structured illumination micrographs of 10 different *Chlamydia* infected HeLa cells **(H)** with ~2 ROI from each picture [Mean of Overlap (R) was 0.863 with ±SD of 0.022; *n* = 1. Mean of Pearson's overlap coefficient (Rr) was 0.012 with ±SD of 0.039; *n* = 1]. Scale bars in all panels, 10 μM.

Figure S5 Functional interaction of CPAF and PI15. **(A)** CPAF zymogen co-immunoprecipitates with PI15. Co-IP experiments were carried out using T-REx-293 cells that inducibly express PI15-Flag. Cells were induced with 0.1 μg/ml of AHT for 48 h and then infected with a CPAF wildtype (CPAF+) and CPAF mutant (CPAF-) strain, for another 24 h. Total cellular lysates were prepared and used for Co-IP using commercial Flag beads. **(B)** siRNA-mediated PI15 silencing inhibits active CPAF formation. PI15 expression was silenced in HeLa cells by siRNAs (siPI15). Control siRNA (siControl) was used for comparison. Proteolytic effect of potential cytoplasmic CPAF was studied by immunoblotting of cytokeratin 8 (CK8) under stringent denaturing conditions. ^*^ non-specific protein bands.

Figure S6 Recombinant PI15 protein preparation. Recombinant PI15 proteins were purified from *E. coli*. Different concentrations of PI15 proteins were separated on a 12% PAGE gel and stained with Coomassie stain **(left)** or immunoblotted using an antibody against PI15 **(right)**.

Video 1 3D reconstruction of PI15-CPAF co-staining. Lateral rotation of the 3D projection around Y of *C. trachomatis* infected HeLa cells stained with CPAF (red channel), PI15 (green channel) and DNA is stained with DAPI (blue channel). Images were acquired in Z-stack on a Leica TCS SP5 confocal platform after fixing and reconstructed from the stacks using the 3D Viewer plugin from FIJI.

Video 2 3D reconstruction from a coronal section of PI15-CPAF co-staining (The cell in entirety is seen in Video 1). Lateral rotation of the 3D projection around Y of *C. trachomatis* infected HeLa cells stained with CPAF (red channel), PI15 (green channel) and DNA is stained with DAPI (blue channel). Images were acquired in Z-stack on a Leica TCS SP5 confocal platform after fixing and reconstructed from the stacks using the 3D Viewer plugin from FIJI.

Videos 3 and 4 3D reconstruction from a coronal section of PI15-CPAF co-staining. Diagonal rotation of the 3D projection around Y of *C. trachomatis* infected HeLa cells stained with CPAF (red channel), PI15 (green channel) and DNA is stained with DAPI (blue channel). Images were acquired in Z-stack on a Leica TCS SP5 confocal platform after fixing and reconstructed from the stacks using the 3D Viewer plugin from FIJI.

## References

[B1] Atwood C. S. Perry G. Zeng H. Kato Y. Jones W. D. Ling K. Q.. (2004). Copper mediates dityrosine cross-linking of Alzheimer's amyloid-beta. Biochemistry 43, 560–568. 10.1021/bi0358824 14717612

[B2] Bavoil P. M. Byrne G. I. (2014). Analysis of CPAF mutants: new functions, new questions (the ins and outs of a chlamydial protease). Pathog. Dis. 71, 287–291. 10.1111/2049-632X.12194 24942261 PMC5914542

[B3] Betts-Hampikian H. J. Fields K. A. (2010). The chlamydial type III secretion mechanism: revealing cracks in a tough Nut. Front. Microbiol. 1:114. 10.3389/fmicb.2010.00114 21738522 PMC3125583

[B4] Bolte S. Cordelières F. P. (2006). A guided tour into subcellular colocalization analysis in light microscopy. J. Microsc. 224(Pt 3), 213–232. 10.1111/j.1365-2818.2006.01706.x 17210054

[B5] Chen A. L. Johnson K. A. Lee J. K. Sütterlin C. Tan M. (2012). CPAF: a Chlamydial protease in search of an authentic substrate. PLoS Pathog. 8:e1002842. 10.1371/journal.ppat.1002842 22876181 PMC3410858

[B6] Chen D. Chai J. Hart P. J. Zhong G. (2009). Identifying catalytic residues in CPAF, a chlamydia-secreted protease. Arch. Biochem. Biophys. 485, 16–23. 10.1016/j.abb.2009.01.014 19388144 PMC2768414

[B7] Chen D. Lei L. Flores R. Huang Z. Wu Z. Chai J.. (2010a). Autoprocessing and self-activation of the secreted protease CPAF in Chlamydia-infected cells. Microb. Pathog. 49, 164–173. 10.1016/j.micpath.2010.05.008 20510344 PMC2917482

[B8] Chen D. Lei L. Lu C. Flores R. DeLisa M. P. Roberts T. C.. (2010b). Secretion of the chlamydial virulence factor CPAF requires the Sec-dependent pathway. Microbiology (Reading, Engl) 156(Pt 10), 3031–3040. 10.1099/mic.0.040527-0 20522495 PMC3068695

[B9] Chowdhury S. R. Reimer A. Sharan M. Kozjak-Pavlovic V. Eulalio A. Prysty B. K.. (2017). Chlamydia preserves the mitochondrial network via a microRNA-dependent inhibition of fission. J. Cell Biol. 216, 1071–1089. 10.1083/jcb.201608063 28330939 PMC5379946

[B10] Christian J. Vier J. Paschen S. A. Hacker G. (2010). Cleavage of the NF- B family protein p65/RelA by the Chlamydial Protease-like Activity Factor (CPAF) impairs proinflammatory signaling in cells infected with chlamydiae. J. Biol. Chem. 285, 41320–41327. 10.1074/jbc.M110.152280 21041296 PMC3009857

[B11] Dong F. Sharma J. Xiao Y. Zhong Y. Zhong G. (2004). Intramolecular dimerization is required for the chlamydia-secreted protease CPAF to degrade host transcriptional factors. Infect. Immun. 72, 3869–3875. 10.1128/IAI.72.7.3869-3875.2004 15213129 PMC427400

[B12] Fischer A. Harrison K. S. Ramirez Y. Auer D. Chowdhury S. R. Prusty B. K.. (2017). Chlamydia trachomatis-containing vacuole serves as deubiquitination platform to stabilize Mcl-1 and to interfere with host defense. Elife 6:e21465. 10.7554/eLife.21465 28347402 PMC5370187

[B13] Fischer A. Rudel T. (2016). Subversion of cell-autonomous host defense by chlamydia infection. Curr. Top. Microbiol. Immunol. [Epub ahead of print]. 10.1007/82_2016_13 27169422

[B14] Gibbs G. Roelants K. O'Bryan M. (2008). The CAP superfamily: cysteine-rich secretory proteins, antigen 5, and pathogenesis-related 1 proteins–roles in reproduction, cancer, and immune defense. Endocr. Rev. 29, 865–897. 10.1210/er.2008-0032 18824526

[B15] Hackstadt T. Scidmore M. A. Rockey D. D. (1995). Lipid metabolism in *Chlamydia trachomatis*-infected cells: directed trafficking of Golgi-derived sphingolipids to the chlamydial inclusion. Proc. Natl. Acad. Sci. U.S.A. 92, 4877–4881. 10.1073/pnas.92.11.4877 7761416 PMC41810

[B16] Herweg J. A. Hansmeier N. Otto A. Geffken A. C. Subbarayal P. Prusty B. K.. (2015). Purification and proteomics of pathogen-modified vacuoles and membranes. Front. Cell. Infect. Microbiol. 5:48. 10.3389/fcimb.2015.00048 26082896 PMC4451638

[B17] Heuer D. Rejman Lipinski A. Machuy N. Karlas A. Wehrens A. Siedler F.. (2009). Chlamydia causes fragmentation of the Golgi compartment to ensure reproduction. Nature 457, 731–735. 10.1038/nature07578 19060882

[B18] Huang Z. Feng Y. Chen D. Wu X. Huang S. Wang X.. (2008). Structural basis for activation and inhibition of the secreted chlamydia protease CPAF. Cell Host Microbe 4, 529–542. 10.1016/j.chom.2008.10.005 19064254

[B19] Jorgensen I. Bednar M. M. Amin V. Davis B. K. Ting J. P. McCafferty D. G.. (2011). The Chlamydia protease CPAF regulates host and bacterial proteins to maintain pathogen vacuole integrity and promote virulence. Cell Host Microbe 10, 21–32. 10.1016/j.chom.2011.06.008 21767809 PMC3147293

[B20] Kaplan F. Ledoux P. Kassamali F. Q. Gagnon S. Post M. Koehler D.. (1999). A novel developmentally regulated gene in lung mesenchyme: homology to a tumor-derived trypsin inhibitor. Am. J. Physiol. 276(6 Pt 1), L1027–L1036. 10.1152/ajplung.1999.276.6.L1027 10362728

[B21] Kokes M. Valdivia R. H. (2015). Differential translocation of host cellular materials into the *Chlamydia trachomatis* inclusion lumen during chemical fixation. PLoS ONE 10:e0139153. 10.1371/journal.pone.0139153 26426122 PMC4591358

[B22] Koshikawa N. Nakamura T. Tsuchiya N. Isaji M. Yasumitsu H. Umeda M.. (1996). Purification and identification of a novel and four known serine proteinase inhibitors secreted by human glioblastoma cells. J. Biochem. 119, 334–339. 10.1093/oxfordjournals.jbchem.a021244 8882727

[B23] Paschen S. A. Christian J. G. Vier J. Schmidt F. Walch A. Ojcius D. M.. (2008). Cytopathicity of Chlamydia is largely reproduced by expression of a single chlamydial protease. J. Cell Biol. 182, 117–127. 10.1083/jcb.200804023 18625845 PMC2447887

[B24] Pirbhai M. Dong F. Zhong Y. Pan K. Z. Zhong G. (2006). The secreted protease factor CPAF is responsible for degrading pro-apoptotic BH3-only proteins in *Chlamydia trachomatis*-infected cells. J. Biol. Chem. 281, 31495–31501. 10.1074/jbc.M602796200 16940052

[B25] Prusty B. K. Böhme L. Bergmann B. Siegl C. Krause E. Mehlitz A.. (2012). Imbalanced oxidative stress causes chlamydial persistence during non-productive human herpes virus co-infection. PLoS ONE 7:e47427. 10.1371/journal.pone.0047427 23077614 PMC3471814

[B26] Prusty B. K. Siegl C. Hauck P. Hain J. Korhonen S. J. Hiltunen-Back E.. (2013). *Chlamydia trachomatis* infection induces replication of latent HHV-6. PLoS ONE 8:e61400. 10.1371/journal.pone.0061400 23620749 PMC3631192

[B27] Rajalingam K. Sharma M. Lohmann C. Oswald M. Thieck O. Froelich C. J.. (2008). Mcl-1 is a key regulator of apoptosis resistance in *Chlamydia trachomatis*-infected cells. PLoS ONE 3:e3102. 10.1371/journal.pone.0003102 18769617 PMC2518856

[B28] Schindelin J. Arganda-Carreras I. Frise E. Kaynig V. Longair M. Pietzsch T.. (2012). Fiji: an open-source platform for biological-image analysis. Nat. Methods 9, 676–682. 10.1038/nmeth.2019 22743772 PMC3855844

[B29] Shaw A. C. Vandahl B. B. Larsen M. R. Roepstorff P. Gevaert K. Vandekerckhove J.. (2002). Characterization of a secreted Chlamydia protease. Cell. Microbiol. 4, 411–424. 10.1046/j.1462-5822.2002.00200.x 12102687

[B30] Smith D. M. Collins-Racie L. A. Marigo V. A. Roberts D. J. Davis N. M. Hartmann C.. (2001). Cloning and expression of a novel cysteine-rich secreted protein family member expressed in thyroid and pancreatic mesoderm within the chicken embryo. Mech. Dev. 102, 223–226. 10.1016/S0925-4773(01)00293-3 11287197

[B31] Snavely E. A. Kokes M. Dunn J. D. Saka H. A. Nguyen B. D. Bastidas R. J.. (2014). Reassessing the role of the secreted protease CPAF in *Chlamydia trachomatis* infection through genetic approaches. Pathog. Dis. 71, 336–351. 10.1111/2049-632X.12179 24838663 PMC4270368

[B32] Takemoto M. He L. Norlin J. Patrakka J. Xiao Z. Petrova T.. (2006). Large-scale identification of genes implicated in kidney glomerulus development and function. EMBO J. 25, 1160–1174. 10.1038/sj.emboj.7601014 16498405 PMC1409724

[B33] Tang L. Chen J. Zhou Z. Yu P. Yang Z. Zhong G. (2015). Chlamydia-secreted protease CPAF degrades host antimicrobial peptides. Microbes Infect. 17, 402–408. 10.1016/j.micinf.2015.02.005 25752416

[B34] Wang J. Frohlich K. M. Buckner L. Quayle A. J. Luo M. Feng X.. (2011). Altered protein secretion of *Chlamydia trachomatis* in persistently infected human endocervical epithelial cells. Microbiology 157(Pt 10), 2759–2771. 10.1099/mic.0.044917-0 21737500 PMC3353392

[B35] Wu X. Lei L. Gong S. Chen D. Flores R. Zhong G. (2011). The chlamydial periplasmic stress response serine protease cHtrA is secreted into host cell cytosol. BMC Microbiol. 11:87. 10.1186/1471-2180-11-87 21527029 PMC3107777

[B36] Yamakawa T. Miyata S. Ogawa N. Koshikawa N. Yasumitsu H. Kanamori T.. (1998). cDNA cloning of a novel trypsin inhibitor with similarity to pathogenesis-related proteins, and its frequent expression in human brain cancer cells. Biochim. Biophys. Acta 1395, 202–208. 10.1016/S0167-4781(97)00149-8 9473672

[B37] Yang Z. Tang L. Sun X. Chai J. Zhong G. (2015). Characterization of CPAF critical residues and secretion during *Chlamydia trachomatis* infection. Infect. Immun. 83, 2234–2241. 10.1128/IAI.00275-15 25776755 PMC4432759

[B38] Zhong G. Fan P. Ji H. Dong F. Huang Y. (2001). Identification of a chlamydial protease-like activity factor responsible for the degradation of host transcription factors. J. Exp. Med. 193, 935–942. 10.1084/jem.193.8.935 11304554 PMC2193410

