# Supplementary figures and images for: Peptidase Inhibitor 15 (PI15) Regulates Chlamydial CPAF Activity
Source: Front Cell Infect Microbiol. 2018 May 30;8:183. doi: 10.3389/fcimb.2018.00183 (PMC5989220; doi:10.3389/fcimb.2018.00183)

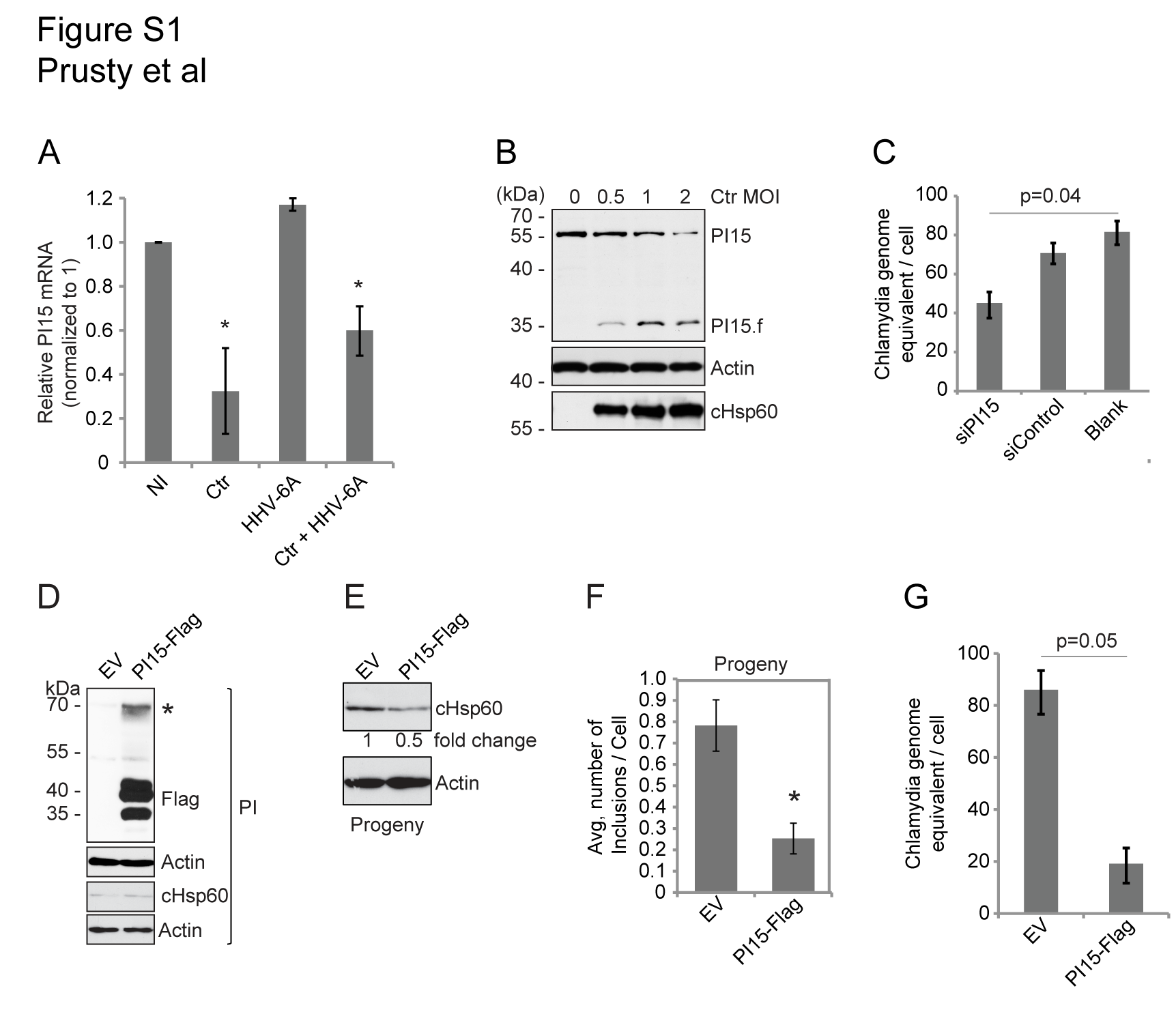

Supplement: Figure S1 — PI15 regulation and its effect on chlamydial growth.(A) PI15 is differentially regulated at transcriptional level during Chlamydia and /or HHV-6A infection. HeLa cells were infected with either C. trachomatis or HHV-6A or both for 24 h. Total RNA was extracted and was used for quantitative reverse transcriptase PCR to analyze PI15 mRNA level. hpi, hours post infection. Data represents ± SEM from 3 independent experiments. * ≤ 0.05. Ctr, Chlamydia; NI, non-infected control. (B) PI15 cleavage was directly correlated with chlamydial load. HeLa cells were infected with C. trachomatis for 36 h with different MOI of infection. PI15 expression was studied by immunostaining. (C) siRNA-mediated silencing of PI15 inhibits chlamydial progeny formation as quantified by qPCR. HeLa cells were transfected with siRNA against human PI15 for 72 h and were subsequently infected with C. trachomatis for another 36 h. Scrambled siRNA were transfected as control. Secondary infection was carried out by applying lysed primary infected cells to fresh HeLa cells to determine Chlamydia growth and progeny formation upon PI15 silencing. Total cellular DNA was extracted from infected HeLa cells 36 hpi and was quantified by qPCR. Chlamydial genome equivalent was normalized with cellular genome equivalent to finally calculate genome equivalents per cell. Data represent ± SEM from 3 independent experiments. (D–G) Transient over-expression of PI15 inhibits chlamydial growth. (D) Flag-tagged PI15 was transiently over-expressed in 293T cells for 24 h. Empty vector (EV) transfection was used as a control. 24 h post transfection, cells were infected with Chlamydia for another 24 h. Immunoblotting was carried out to validate PI15 over-expression. cHsp60 was tested to compare chlamydial growth during primary infection (PI). *, Unknown protein. (E) Secondary infectivity assay were carried out to determine infectious progeny formation. cHsp60 levels were compared in progeny infected cells (F). Average number [file Image_1.TIF]

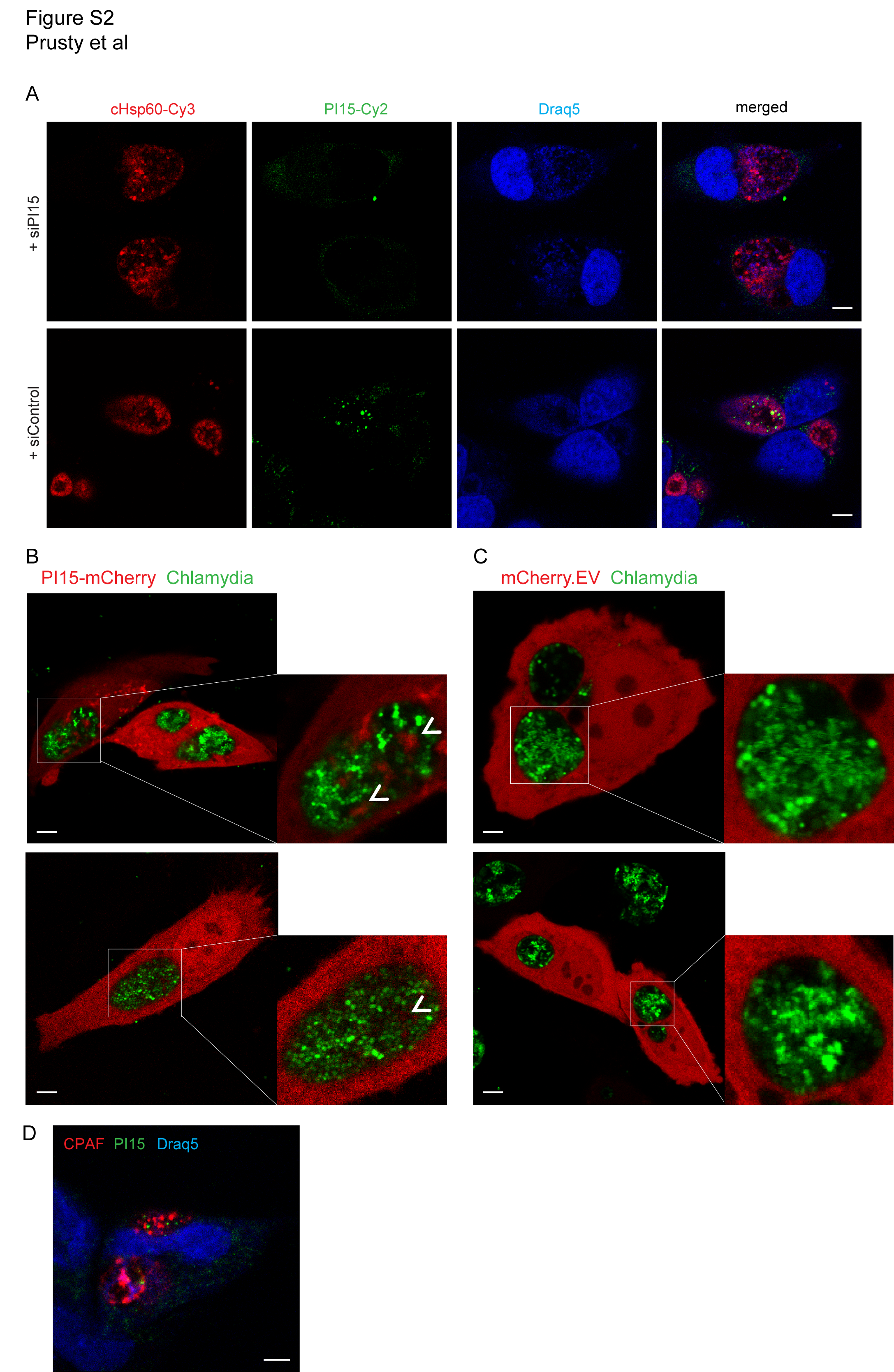

Supplement: Figure S2 — PI15 localizes within the chlamydial inclusion lumen. (A) Validation of PI15-specific antibody staining by RNAi. HeLa cells were either transfected with siRNAs against PI15 or control siRNAs. 72 h post transfection, cells were infected with C. trachomatis. Cells were immunostained using PI15 or Chlamydia-specific Hsp60 (cHsp60) antibodies. Draq5 was used to stain DNA. Individual channel images are presented. (B,C) Live cell imaging was carried out in HeLa cells transiently transfected with mCherry-PI15 (B) or an empty mCherry vector (C). 6 h post transfection cells were infected with GFP-expressing Chlamydia for another 24 h. Cellular localization of mCherry protein was carried out in live cells using a Leica SP5 microscope. (D) PI15 is localized within the inclusion during C. trachomatis serovar D infection. HeLa cells were infected with C. trachomatis serovar D for 24 h. Cells were immunostained using PI15 or CPAF antibodies. Draq5 was used to stain DNA. Scale bars in all panels, 10 μM. [file Image_2.TIFF]

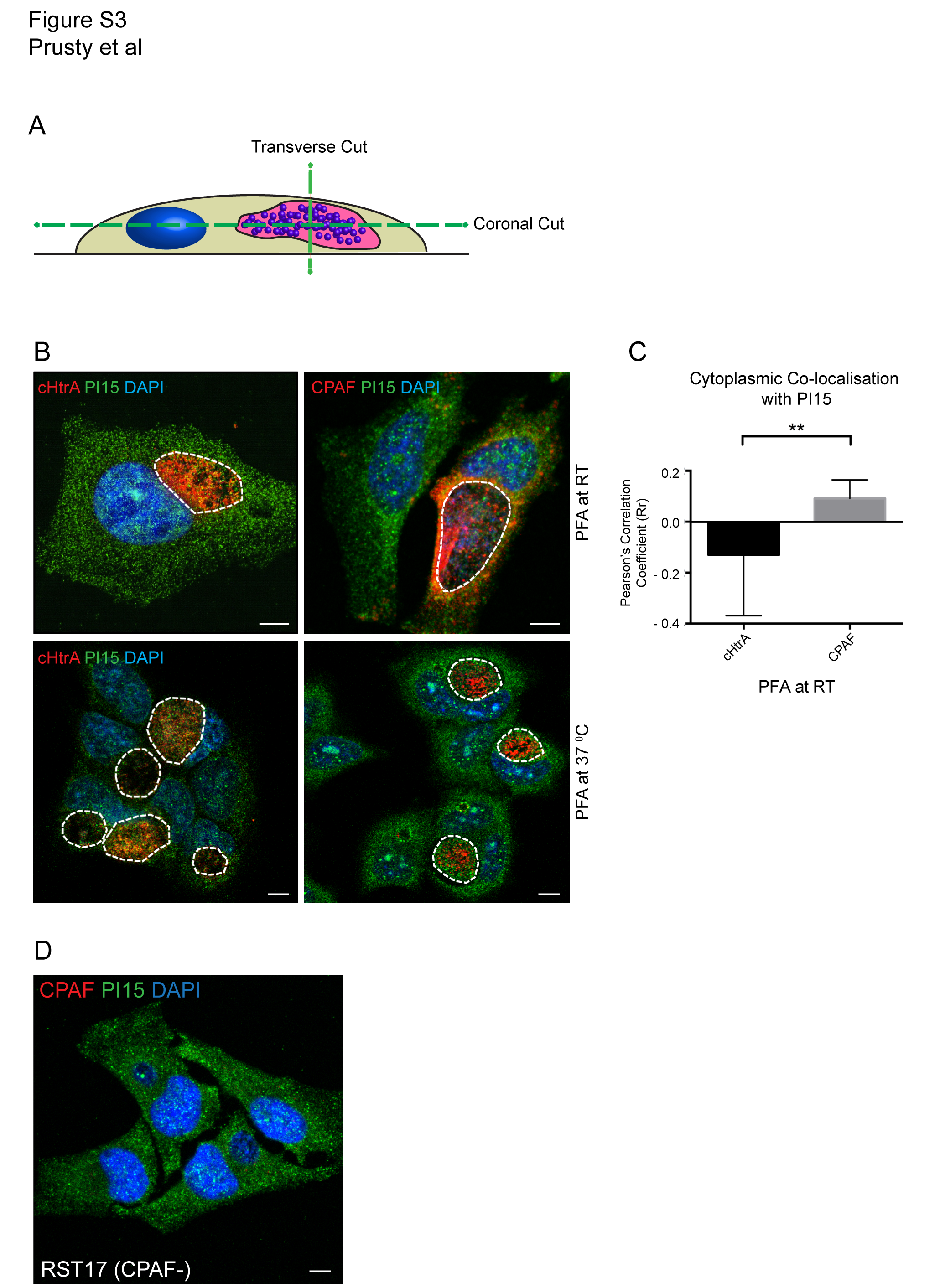

Supplement: Figure S3 — Comparison of intra-cellular CPAF localization by different fixation methods. (A) Schematic diagram showing different planes of sectioning of images used for the study of PI15-CPAF co-localization. (B) Confocal images from CPAF or cHtrA stained Chlamydia infected cells fixed with PFA either at room temperature (RT) or at 37°C. DAPI staining was used to stain DNA. Inclusions are marked with white dotted lines. (C) Cytoplasmic co-localization of CPAF or cHtrA with PI15 was quantified and Pearson's co-efficient was plotted as explained before. ** ≤ 0.005. (D) CPAF specific staining was verified by infecting HeLa cells with a mutant strain of Chlamydia that does not express CPAF and by using immunostaining. Scale bars in all panels, 10 μM. [file Image_3.TIFF]

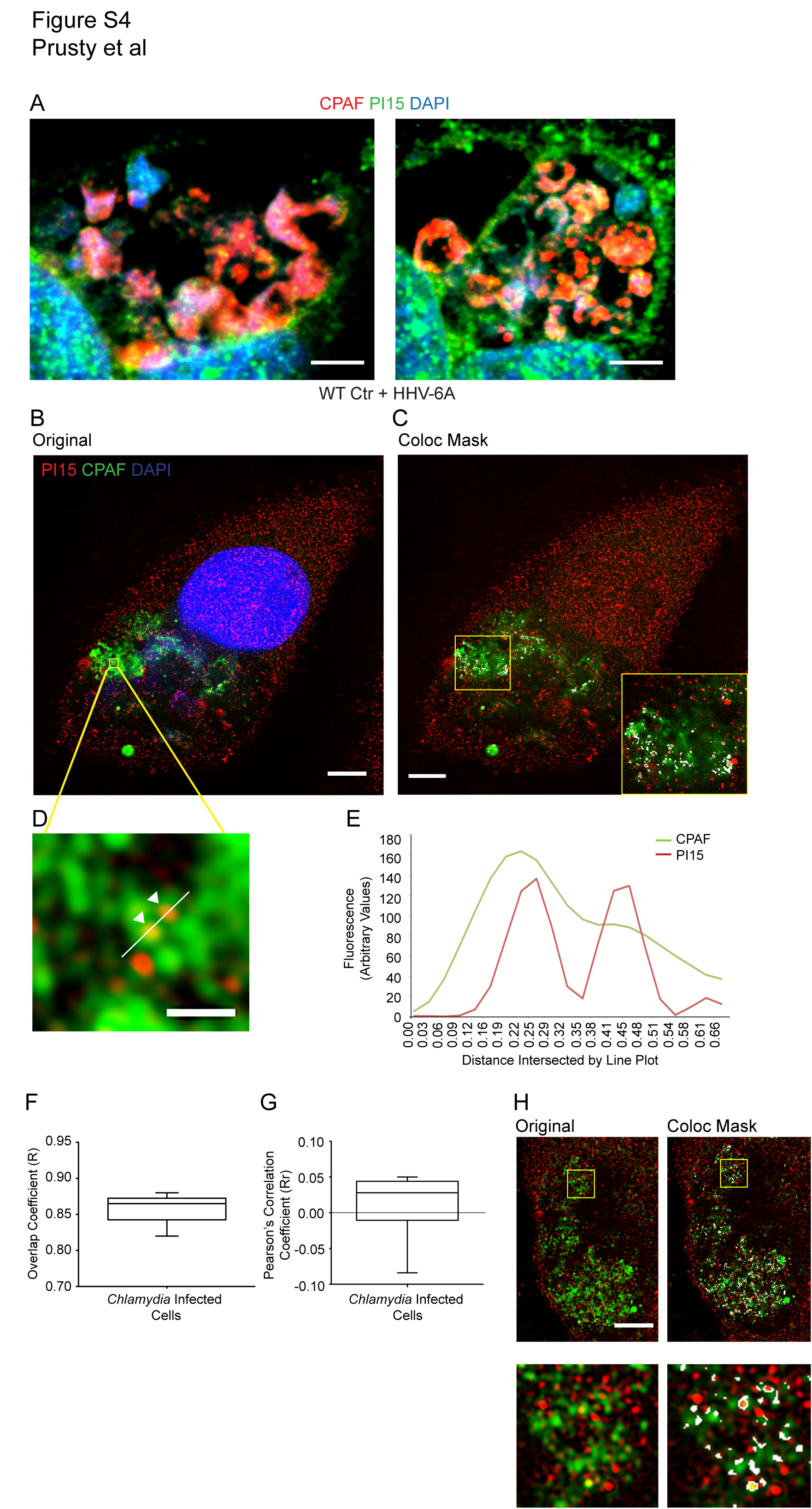

Supplement: Figure S4 — CQuantitative analysis of PI15 and CPAF co-localization within inclusions of persistent Chlamydia. (A) Confocal images showing CPAF and PI15 co-localization within two representative persistent chlamydial inclusions infected with wild type Chlamydia (WT Ctr) and HHV-6A. (B) Structured illumination micrograph of a Chlamydia infected HeLa cell immunostained for CPAF (green) and PI15 (red). The nucleus was stained with DAPI (blue). (C) Co-localisation Mask (coloc mask) was generated using the COLOC2 plugin from FIJI. The white regions illustrate the areas of overlaps between CPAF and PI15 observed only within the chlamydial inclusion. (D) The inset from (A) was used to generate a signal overlap graph (E) by plotting arbitrary fluorescence values against distance traversed by the white line in (C). (F) Overlap values (percentage of signal overlap between two particles) and (G) Pearson's overlap coefficient (Rr; representing the degree of overlap between two groups of particles in the image) were obtained by processing Structured illumination micrographs of 10 different Chlamydia infected HeLa cells (H) with ~2 ROI from each picture [Mean of Overlap (R) was 0.863 with ±SD of 0.022; n = 1. Mean of Pearson's overlap coefficient (Rr) was 0.012 with ±SD of 0.039; n = 1]. Scale bars in all panels, 10 μM. [file Image_4.TIFF]

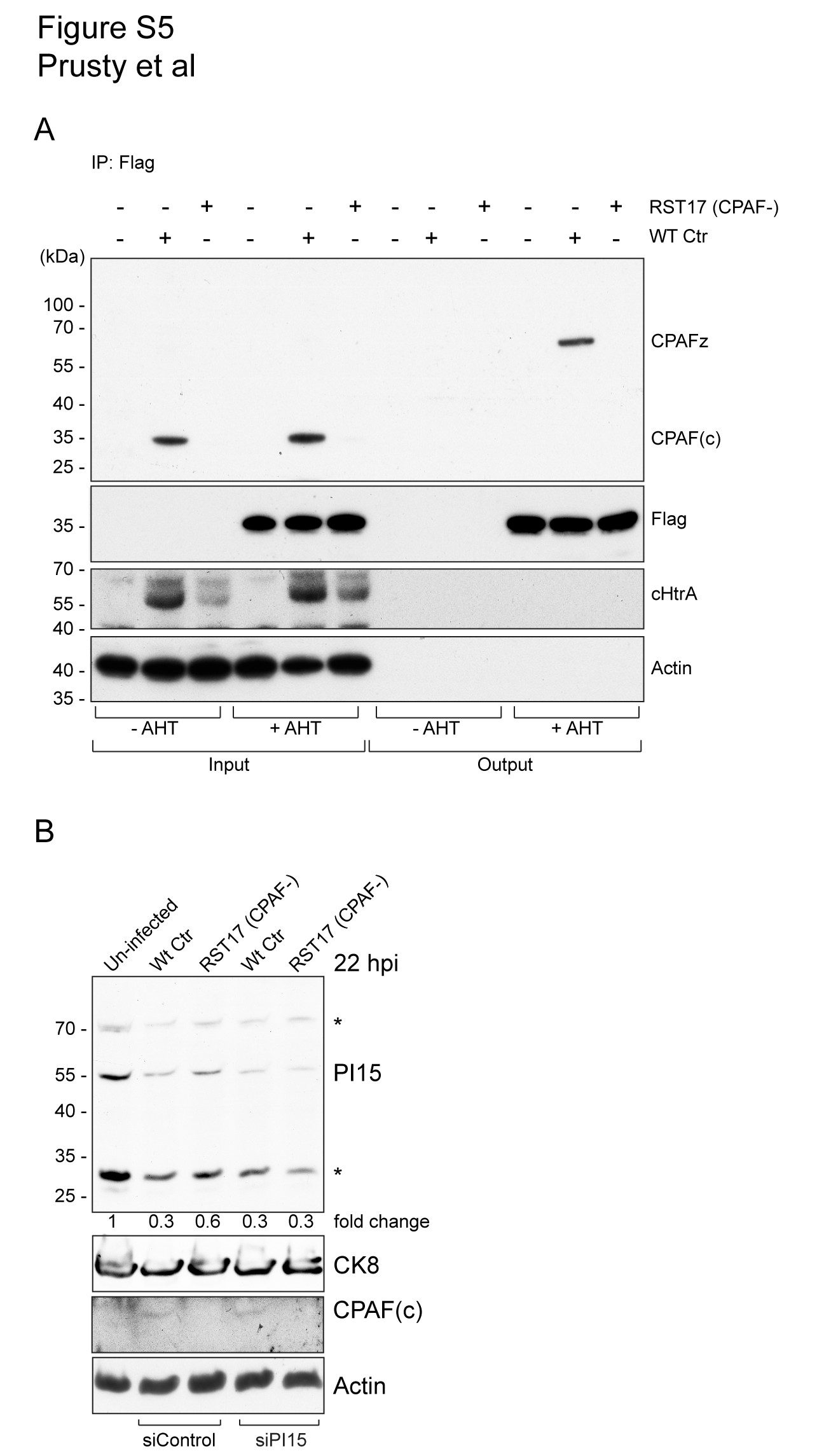

Supplement: Figure S5 — Functional interaction of CPAF and PI15. (A) CPAF zymogen co-immunoprecipitates with PI15. Co-IP experiments were carried out using T-REx-293 cells that inducibly express PI15-Flag. Cells were induced with 0.1 μg/ml of AHT for 48 h and then infected with a CPAF wildtype (CPAF+) and CPAF mutant (CPAF-) strain, for another 24 h. Total cellular lysates were prepared and used for Co-IP using commercial Flag beads. (B) siRNA-mediated PI15 silencing inhibits active CPAF formation. PI15 expression was silenced in HeLa cells by siRNAs (siPI15). Control siRNA (siControl) was used for comparison. Proteolytic effect of potential cytoplasmic CPAF was studied by immunoblotting of cytokeratin 8 (CK8) under stringent denaturing conditions. * non-specific protein bands. [file Image_5.TIF]

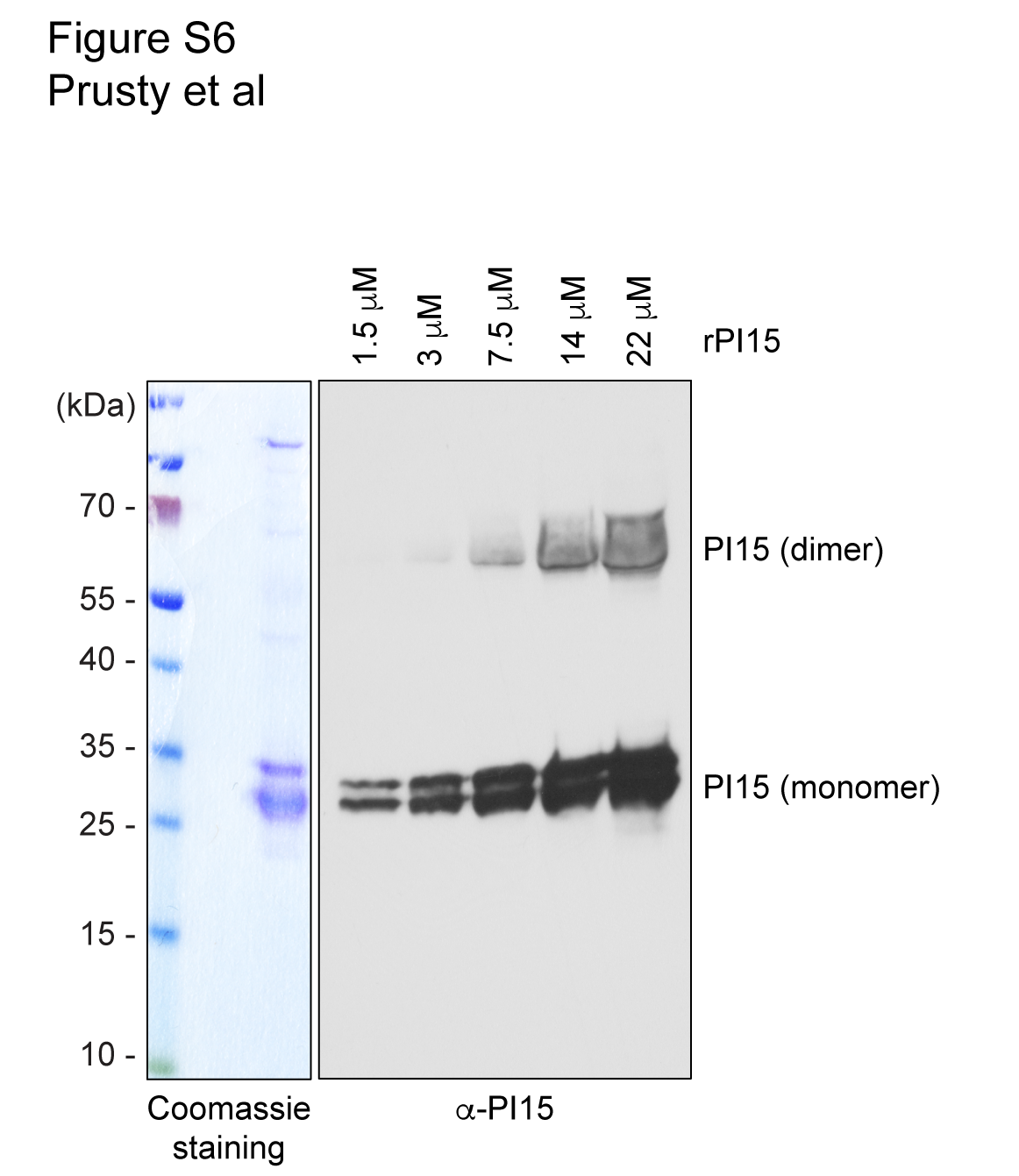

Supplement: Figure S6 — Recombinant PI15 protein preparation. Recombinant PI15 proteins were purified from E. coli. Different concentrations of PI15 proteins were separated on a 12% PAGE gel and stained with Coomassie stain (left) or immunoblotted using an antibody against PI15 (right). [file Image_6.TIF]
